# Comparative Genome Analyses Reveal Distinct Structure in the Saltwater Crocodile MHC

**DOI:** 10.1371/journal.pone.0114631

**Published:** 2014-12-11

**Authors:** Weerachai Jaratlerdsiri, Janine Deakin, Ricardo M. Godinez, Xueyan Shan, Daniel G. Peterson, Sylvain Marthey, Eric Lyons, Fiona M. McCarthy, Sally R. Isberg, Damien P. Higgins, Amanda Y. Chong, John St John, Travis C. Glenn, David A. Ray, Jaime Gongora

**Affiliations:** 1 Faculty of Veterinary Science, University of Sydney, Sydney, New South Wales 2006, Australia; 2 Evolution Ecology and Genetics, Research School of Biology, Australian National University, Canberra, Australian Capital Territory 2601, Australia; 3 Institute for Applied Ecology, University of Canberra, Canberra, Australian Capital Territory 2601, Australia; 4 Department of Organismic and Evolutionary Biology, Harvard University, Cambridge, Massachusetts 02138, United States of America; 5 Department of Biochemistry, Molecular Biology, Entomology and Plant Pathology, Mississippi State University, Mississippi State, Mississippi 39762, United States of America; 6 Institute for Genomics, Biocomputing and Biotechnology (IGBB), Mississippi State University, Mississippi State, Mississippi 39762, United States of America; 7 Animal Genetics and Integrative Biology, INRA, UMR 1313 Jouy-en-Josas 78352, France; 8 School of Plant Science, University of Arizona, Tucson, Arizona 85721, United States of America; 9 School of Animal and Comparative Biomedical Sciences, University of Arizona, Tucson, Arizona 85721, United States of America; 10 Center for Crocodile Research, P.O. Box 329, Noonamah, Northern Territory 0837, Australia; 11 Department of Biomolecular Engineering, University of California Santa Cruz, Santa Cruz, California 95064, United States of America; 12 Department of Environmental Health Science, University of Georgia, Athens, Georgia 30602, United States of America; 13 Department of Genetics, Harvard Medical School, 77 Louis Pasteur Ave., Boston, Massachusetts 02115, United States of America; Laboratoire de Biologie du Développement de Villefranche-sur-Mer, France

## Abstract

The major histocompatibility complex (MHC) is a dynamic genome region with an essential role in the adaptive immunity of vertebrates, especially antigen presentation. The MHC is generally divided into subregions (classes I, II and III) containing genes of similar function across species, but with different gene number and organisation. Crocodylia (crocodilians) are widely distributed and represent an evolutionary distinct group among higher vertebrates, but the genomic organisation of MHC within this lineage has been largely unexplored. Here, we studied the MHC region of the saltwater crocodile (*Crocodylus porosus*) and compared it with that of other taxa. We characterised genomic clusters encompassing MHC class I and class II genes in the saltwater crocodile based on sequencing of bacterial artificial chromosomes. Six gene clusters spanning ∼452 kb were identified to contain nine MHC class I genes, six MHC class II genes, three TAP genes, and a TRIM gene. These MHC class I and class II genes were in separate scaffold regions and were greater in length (2–6 times longer) than their counterparts in well-studied fowl *B* loci, suggesting that the compaction of avian MHC occurred after the crocodilian-avian split. Comparative analyses between the saltwater crocodile MHC and that from the alligator and gharial showed large syntenic areas (>80% identity) with similar gene order. Comparisons with other vertebrates showed that the saltwater crocodile had MHC class I genes located along with TAP, consistent with birds studied. Linkage between MHC class I and TRIM39 observed in the saltwater crocodile resembled MHC in eutherians compared, but absent in avian MHC, suggesting that the saltwater crocodile MHC appears to have gene organisation intermediate between these two lineages. These observations suggest that the structure of the saltwater crocodile MHC, and other crocodilians, can help determine the MHC that was present in the ancestors of archosaurs.

## Introduction

The major histocompatibility complex (MHC) is one of the most dynamic genomic regions involved in self/non-self recognition and defense against pathogens in jawed vertebrates [1], [2]. The MHC typically consists of three regions: class I, class II, and class III [3]. The MHC class I region is occupied by genes predominantly encoding for proteins that present intracytoplasmic (e.g. viral) antigens for the activation of cytotoxic T-cells (CD8^+^), whereas the MHC class II region consists of genes responsible for presentation of phagocytosed antigens from extracellular pathogens to helper T-cells (CD4^+^). The MHC class III region contains genes with a functional role in innate (non-specific) immunity, inflammation, and regulation of the immune response [4]. Gene content and orientation of MHC regions has diverged throughout vertebrate evolution as species evolved and adapted to pathogenic pressures in their environment [5]. To date, MHC has been well-characterised in more than 20 species and four major vertebrate groups, including fish [6], [7], amphibians [8], birds [9]–[13], and mammals [5], [14], [15]. Assessment of non-avian reptile MHC is needed for comparisons of gene gain and loss, as well as gene rearrangement (i.e. insertions, deletions, inversion, and gene conversion) between this lineage and others, as it will be crucial to understand species adaption to environmental challenges and provide background information on evolutionary potentials of the lineage to fight against pathogens [16]–[18].

MHC gene content and organisation varies among vertebrate groups. In teleost fish, MHC genes are known to spread across multiple chromosomes [19], [20]. For instance, zebrafish MHC class II genes have been reported to split into two or more unlinked loci [21], [22]. Some fish, such as the Atlantic cod (*Gadus morhua*) and pipefish (*Syngnathus typhle*), are found to have lost MHC class II genes [23], [24]. In contrast, MHC in most eutherian mammals consists of syntenic clusters of class I, II and III regions, which includes the large gene-dense region with MHC class III genes positioned in-between class I and class II. Mammals are found to have complex and varying numbers of MHC class I and II genes [5], [15]. For instance, the human MHC (human leukocyte antigen, HLA) consists of six class I genes (*HLA-A, -B, -C, -E, -F* and -*G*), and five class II gene groups (DP, DO, DM, DQ and DR), with different numbers of genes per group within an individual [25]. In addition, eutherians have framework genes within the MHC class I region, which are thought to play a role in duplication and insertion events [26], and antigen processing genes within the MHC class II region, which are responsible for peptide loading into MHC molecules [27].

Birds are the closest phylogenetic group to Crocodylia, together they form a group known as archosaurs [28]. Complete sequences of avian MHC have been documented to have exceptional gene content and organisation. The chicken (*Gallus gallus*) has one of the smallest core MHC regions (*B* locus) spanning 92 kb in length and containing only 19 genes (from *B-BTN1* to *CRP21* gene), most of which have a human MHC counterpart [9]. Although Shiina et al. [29] have revealed an additional 25 genes beyond the 92-kb core (in total, 46 genes within 242 kb), most have uncertain functions in immunity, such as tripartite motif (TRIM; framework gene), C-type lectin (Blec) and immunoglobulin superfamily type (BG) genes. The non-core regions do not contain antigen presentation genes (MHC class I and II). The *B* locus is thought to be the main functional MHC region, with a small number of MHC genes necessary for adaptive immune responses (known as ‘minimum essential MHC’). The *B* locus consists of two antigen presentation genes of the MHC class I region (*BF1* and *BF2*), and two of the MHC class II region (*BLB1* and *BLB2*) [9]. This MHC structure results from an enhanced degree of gene loss and size reduction in the chicken genome [30]. The chicken MHC differs from the eutherian mammal MHC, not only in its size, but also in the orientation of its three MHC regions, whereby the class I region is surrounded by class II and III [29]. Other differences are the position of the antigen processing genes (TAP1 and TAP2) that are found in the MHC class I region of the chicken *B* locus [9], [15], and the presence of putative natural killer cell receptor-related genes in the chicken [9]. It remains uncertain as to what extent this simple MHC structure is generalised among other related species. The black grouse (*Tetrao tetrix*) and golden pheasant (*Chrysolophus pictus*) show similar MHC simplicity to the 92-kb *B* locus in the chicken, with their MHC cores that contain 19 genes (88 kb) [31] and 20 genes (97 kb) [13], respectively. However, genomic studies in other birds, including the quail (*Coturnix japonica*) and turkey (*Meleagris gallopavo*), have not indicated restriction to a minimum essential MHC in their genomes, despite their consistency in gene organisation with the chicken 92-kb MHC core [10], [12]. The turkey has 34 genes within a 197-kb *B* locus [12] and the quail has 41 genes within a 180-kb *B* locus [10]. All of these avian species belong to a single order (Galliformes), so the role of gene loss versus duplication within the MHC of these lineages and their role in adaptation to environmental challenges [32] requires additional work.

Characterisation of the MHC among distantly related species provides a valuable resource to investigate the evolution of MHC among vertebrates. Recent comparative genomic analyses of fish, amphibians, birds, and mammals have provided a model of MHC evolution, illustrating structural transition of the MHC from a simple ancestral form to a complex and interactive form [1], [5], [15]. The simple MHC structure, whereby class I and class II genes are located together at one end of the region along with antigen processing genes, is suggested to be an ancestral form of the MHC. This structure is found commonly in non-mammalian species, such as nurse sharks (*Ginglymostoma cirratum*) [33], [34], frogs (*Xenopus tropicalis*) [8], and chicken [9], as well as in marsupials, such as opossums (*Monodelphis domestica*) [15] and tammar wallabies (*Macropus eugenii*) [35]. Furthermore, large and complex MHC regions have been observed thus far in mammals [5] and the amphibian *Xenopus* [8]. The MHC class I–III–II structure described above in eutherian mammals appears to have evolved relatively recently; MHC class I genes must have relocated across the MHC class III region on the opposite side of the MHC class II region and interspersed between the framework genes. It is estimated that this eutherian MHC structure occurred after the divergence of marsupials and eutherians [15], which is estimated to be 161–185 million years ago (MYA) [36].

Non-avian reptiles have emerged since the lines leading to birds and mammals diverged more than 300 MYA [36] and consist of diverse orders with several thousands of species, making them ideal for comparative MHC studies. To date, knowledge of MHC in non-avian reptiles is limited, creating difficulties in making MHC comparisons with other vertebrates. Only one genomic comparative study has been performed in non-avian reptiles (Godinez et al., manuscript in revision), which is used as the framework for comparative analyses of the current analysis. The large and complex MHC in the green anole (*Anolis carolinensis*) contains more than 300 genes and it is shown to have a MHC class III region closely linked to a framework class I region and a cluster of MHC class I genes. The green anole MHC also shows close linkage between the MHC class I and an antigen processing gene, as well as close proximity between MHC class I and II genes (Godinez et al., manuscript in revision). Most recently, genomic resources of three species of Crocodylia (the saltwater crocodile, *Crocodylus porosus*; the American alligator, *Alligator mississippiensis*; and the Indian gharial, *Gavialis gangeticus*) have been developed by the International Crocodilian Genomes Working Group (ICGWG). These resources include bacterial artificial chromosome (BAC) libraries [37] and whole genome sequencing and gene annotation efforts [28], [38], which have allowed assembly and annotation of the MHC in the three species. Wan et al. [39] have recently reported the 2.3-Gb genome of the Chinese alligator (*Alligator sinensis*), but the core MHC region was not described in that study. Although a number of MHC class I genes have been predicted in the Chinese alligator genome [39], the MHC sequences were not made publicly available at the time of publication. The Order Crocodylia is the best extant outgroup for comparison with avian genomes [28]. As such, the MHC in Crocodylia allows us to assess whether the reduction in size and gradual gene loss in avian genomes that is thought to have resulted in the minimalist *B* locus and its variation in fowl also applies to crocodilian genomes. Alternatively, we assess whether a combination of this genome size reduction and independent evolutionary forces have resulted in differences of MHC structure in the Archosauria. Here, we use available genomic resources [40]–[42] to identify BAC clones [37] that contain MHC sequences and assess regions of MHC class I and II based on construction of BAC contigs/scaffolds in the saltwater crocodile.

## Results

### BAC library screening

In total, 103 BACs were identified as positive for the MHC regions, based on results of both primary and secondary screening (S1 Table). Nine BACs that showed very strong signal during the BAC screening, clear PCR bands, and sequences consistent with MHC genes of interest, were then chosen for sequencing and downstream analyses.

### Characterisation of MHC gene clusters in the saltwater crocodile genome

**Figure 1:**
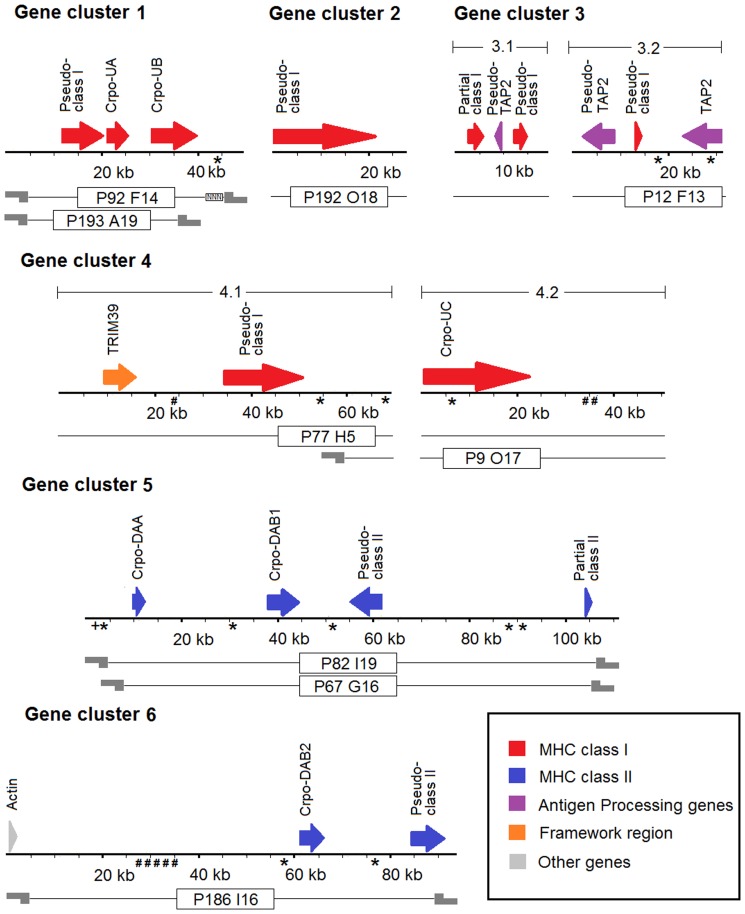
Schematic diagram of six saltwater crocodile gene clusters representing MHC class I and II. Arrows indicate annotated genes and their strands (plus and minus); lines with names in the boxes below the annotation indicate BAC clones corresponding to the MHC gene clusters; and sticky ends show restriction sites of *Hind III* enzyme, and therefore BAC end sequencing. For retrotransposon sequences and endogenous retrovirus (ERV) sequences, asterisks indicate retrotransposon reverse transcriptase (RT) proteins; hashes indicate Gag-Pol precursor polyproteins; and pluses indicate non-LTR retrotransposon LINE-1 (L1).

Six MHC gene clusters encompassing 452,493 bp were characterised. Among these clusters, the following 20 genes and pseudogenes were predicted using transcript-based homology and *ab initio* gene prediction: nine for MHC class I, six for MHC class II, three for antigen-processing genes (transporter 2 associated with antigen processing, TAP2), one for TRIM39 and a single actin pseudogene (Table 1; Fig. 1). Among them, five of MHC class I, two of MHC class II, and a single gene of TAP2 showed in-frame stop codons and/or large deletions of entire/partial exons, indicating that they were putative pseudogenes. Four MHC gene clusters (clusters 2, 3, 4 and 6) contained MHC class I genes, and two other gene clusters (clusters 1 and 5) contained MHC class II genes. All the MHC gene clusters sequenced and assembled in the current study had more than 95% identity to genomic scaffolds (∼374 kb in total) of the saltwater crocodile previously generated by ICGWG [28], and showed either large or complete overlapping fragments with them. Genomic sequences and gene annotations were submitted to the ICGWG (ftp://ftp.crocgenomes.org/pub/) and added to ICGWG genome resources for the saltwater crocodile (v0.2). The gene annotation information was incorporated into gff files and integrated into CrocBase for genome viewing.

**Table 1:** List of annotated MHC gene clusters of saltwater crocodile genome from a total of nine BACs.

Gene cluster	Length (bp)	Scaffold^a^/accession no.	BAC clone ID^b^	Start^c^	End^d^	Strand	Exon	Description
1	49565	scaffold-266	P92 F14 (P)	11961	20285	+	8	Truncated MHC class I antigen, pseudo-class I
		(41015 bp);	P193 A19 (C)	21047	26101	+	9	MHC class I antigen, *Crpo-UA*
		KP118845		30592	>39846	+	>4	MHC class I antigen, *Crpo-UB*
2	28338	scaffold-11053	P192 O18 (P)	26	22403	+	8	MHC class I antigen, pseudo-class I
		(15897 bp);						
		KP118842						
3.1	18621	scaffold-10298	P12 F13(P)	<2746	6082	+	>7	MHC class I antigen, partial class I
		(11851 bp);		9743	8351	−	7	Truncated antigen peptide transporter 2, pseudo-TAP2
		KP118844		12699	14600	+	4	Truncated MHC class I antigen, pseudo-class I
3.2	31246	scaffold-7467	P12 F13 (P)	8733	2687	−	12	Antigen peptide transporter 2 (TAP2) pseudogene,
		(31246 bp);						pseudo-TAP2
		KP118843		13758	14041	+	1	Truncated MHC class I antigen, pseudo-class I
				31195	22602	−	11	Antigen peptide transporter 2 (TAP2)
4.1	69185	scaffold-19650	P9 O17 (P)	8913	15882	+	5	Tripartite motif-containing protein 39-like, TRIM39
		(69203 bp);	P77 H5(P)	34457	50813	+	6	Truncated MHC class I antigen, pseudo-class I
		KP118847						
4.2	50603	KP036996	P9 O17 (P)	352	22617	+	7	MHC class I antigen, *Crpo-UC*
			P77 H5 (P)					
5	111194	scaffold-2257	P82 I19 (C)	9848	12052	+	4	MHC class II alpha chain, *Crpo-DAA*
		(111218 bp);	P67 G16 (C)	37889	43833	+	6	MHC class II beta chain, *Crpo-DAB1*
		KP118841		61987	55155	−	4	MHC class II beta chain, pseudo-class II
				109568	>110298	+	>2	MHC class II beta chain, partial class II
6	93741	scaffold-16192	P186 I16 (C)	1136	1739	+	2	Truncated actin, cytoskeletal 1A-like
		(93750 bp);		61439	66139	+	6	MHC class II beta chain, *Crpo-DAB2*
		KP118846		84792	91595	+	3	Truncated MHC class II beta chain, pseudo-class II

a Genetic scaffolds from whole genome sequencing project of the saltwater crocodile (DA Ray laboratory unmasked v0.2; ftp://ftp.crocgenomes.org/pub/).

b BAC clones from the saltwater crocodile genomic library [37]. Completely and partially sequenced BAC clones are abbreviated as C and P in bracket, respectively.

c,d Sequence range (<or>) is provided if entire length of a gene is uncharacterised.

### Comparative analyses of saltwater crocodile MHC

**Figure 2:**
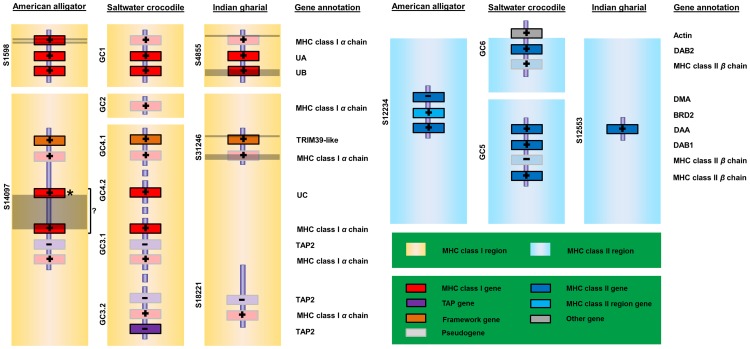
Comparison of the saltwater crocodile, American alligator and Indian gharial MHC class I and II. Scaffold ID is indicated on the left of each genomic scaffold with S (an abbreviation of a scaffold) or GC (an abbreviation of a gene cluster) followed by number. MHC gene clusters identified in the current saltwater crocodile genome assembly are illustrated in Fig. 1. Scaffolds from the American alligator (unmasked v0.2.1, id 19558) and Indian gharial (unmasked v0.2, id 19547) are retrieved from CoGe database. Annotation for each row of genes across these three species is indicated on the last column. Dark areas within the MHC class I region indicates ambiguous sites. Plus and minus signs indicate sequence strand. A question mark suggests uncertainty of identifying a single gene or separate genes of MHC class I due to a sequence gap; asterisks indicate genes of which only *α* or *β* domains are available to assess intact open reading fragments.

Comparisons between the saltwater crocodile MHC characterised here and genome sequences available for the American alligator and Indian gharial showed that their sequences corresponded to two MHC regions, class I and class II (Fig. 2). The MHC class I region contained MHC class I genes, framework genes (TRIM39) and antigen processing genes (TAP), while the MHC class II region contained MHC class II genes and, in the alligator only, a bromodomain containing 2 (BRD2). Twenty of these genes/pseudogenes were characterised in the saltwater crocodile MHC regions, but only eight and ten of them accounting for 40% and 50% of the saltwater crocodile genes identified in this analysis were observed in the gharial and alligator scaffolds, respectively (S2 Table). In addition, three genes/pseudogenes within the MHC class I region of the alligator and four from the gharial homologous region showed small and large regions of ambiguity. These ambiguous nucleotide sites and genes could reflect differences in sequencing methods used in the three species of Crocodylia. Particularly, sequence assembly using whole genome sequencing, such as that used to generate the alligator and gharial sequences, is prone to errors caused by the extensive repetitive DNA content and duplicated fragments in a sequence [43].

Analyses of sequence similarity between MHC sequences showed that three and four scaffolds from the alligator and gharial, respectively, contained sequences conserved with the saltwater crocodile MHC gene clusters (E-value  = 0.0). These scaffolds consisted of the same gene number and order as observed in the saltwater crocodile (Fig. 2; S1 and S2 Figures). For the saltwater crocodile MHC class I, gene cluster 4.2 appeared to occupy the region between gene clusters 3.1 and 4.1 from 5′ to 3′ direction when compared to the arrangement of the alligator scaffold-14097, showing a large MHC class I region consisting of a framework gene, MHC class I genes and antigen processing genes.

**Figure 3:**
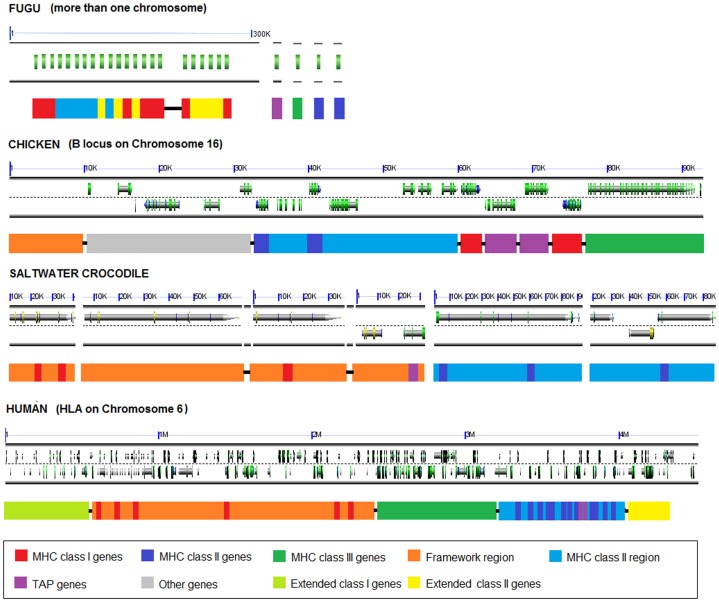
Comparative MHC organisations of the fugu, chicken, saltwater crocodile and human. MHC mapping in the fugu, chicken, and human is generated using data from Clark et al. [87], AL023516 (plus Shiina et al. [29] for a framework region), and NT_007592, respectively. Gene cluster 2 of the saltwater crocodile, where a gene model of coding sequences is absent, is omitted in this figure. Graphics in the first row of each vertebrate represent genes in the MHC based on schematic representation in GEvo [88], where all graphics are automatically created if applicable. Unlinked MHC genes and regions in the fugu and saltwater crocodile presented by the absence of line connection indicate that their order is arbitrary and is not based on the current data. Gray arrows indicate gene models; green arrows indicate protein coding sequences (CDS); blue arrows (on top of gray genes) indicate mRNA; and yellow arrows indicate approximately 50% GC content in codon wobble positions. Scales above the graphics show different sizes of MHC regions in kilo base pairs (K) or mega base pairs (M).

The comparative MHC analysis of the saltwater crocodile, fugu (*Fugu rubripes*), chicken (or red jungle fowl, *Gallus gallus*), and human showed that the present saltwater crocodile genome structure consisted of two independent regions of MHC class I and two of MHC class II (Fig. 3). Based upon the current crocodile sequencing and gene annotation, it is not clear if the crocodile MHC class I and II regions are separated like MHC class I and II regions in the chicken and human, or intermingled like those of the fugu. MHC class I regions of both the saltwater crocodile and chicken showed a close linkage between MHC class I gene and antigen processing gene (TAP), reflecting an associate role of the TAP genes in peptide loading into MHC class I molecules [44]. This was in contrast with the human MHC, where the TAP genes were located closely with MHC class II genes. One difference of MHC organisation between the saltwater crocodile and chicken was linkage between TRIM39 and MHC class I gene in the saltwater crocodile (18,575 bp distant between TRIM39 and MHC class I pseudogene in gene cluster 4.1). In the chicken, it was reported that framework genes, such as TRIM genes, were located 41 kb upstream of the core *B* locus with TRIM-class II-class I-class III orientation [29]. Identifying collinear sets of MHC regions/genes of sequence similarity to infer synteny between the saltwater crocodile and the chicken (plus the human), using SynMap [45], did not show significant large conserved sequences among these comparisons, even though the two species are closely related within Archosauria. It is likely, because the saltwater crocodile compared had much larger MHC regions (452,493 bp in total) and the greater number of MHC class I and II genes/pseudogenes than the fowl *B* locus (92 Kbp). Only a single gene, the saltwater crocodile TAP2, was shown to present a synteny to TAP2 coding sequences of the fowl *B* locus (S3 Figure), suggesting their orthologous relationships between the two species.

### MHC class I genes

#### Gene structure and content

A total of nine MHC class I loci were identified from gene cluster 1 to gene cluster 4 (S4 Figure). Three of these loci were named *Crpo-UA*, *UB* and *UC,* which had intact and complete open reading fragments for all *α* domains responsible for peptide loading. These genes contained between four and nine exons and encoded for proteins that were 408, 293, and 350 amino acids (aa), respectively. The first four exons represented a leader peptide, and three extracellular domains (*α*1, *α*2 and *α*3) while the remaining exons encoded for a transmembrane domain and cytoplasmic tail. All the three genes contained conserved amino acid positions that are well-known for their biological roles in salt bridge-forming, disulfide bridge-forming, N-glycosylation, and CD8^+^ binding, as well as likely peptide-binding of antigen N and C termini. Although it is unclear whether these genes are functional or not, homology searches with transcriptome data from the American alligator showed that the alligator cDNA sequence, Class I transcript had significant matches to *UA* (E-value  = 2e–107, aa identity  = 54%, query coverage  = 80%), *UB* (4e–153, 70%, 98%), and *UC* genes (2e–167, 80%, 80%). This alligator cDNA sequence was found to be expressed in testes, spinal cord, thalamus, and liver. Another MHC class I gene locus in gene cluster 3.1 was partially sequenced from the *α*2 domain to cytoplasmic tail. It was suggested to be putatively functional, because it matched significantly with Class I transcript (E-value  = 1e–124, aa identity  = 81%, query coverage  = 96%) and shared high aa identity to *UB* (88.6%) and *UC* genes (90.2%). The remaining five loci of MHC class I showed truncated features in their open reading fragments, suggesting that they were pseudogenes: one in gene cluster 1 contained deletions in the *α*2 domain; one in gene cluster 2 contained a stop codon in the *α*3 domain; one in gene cluster 3.1 contained a stop codon in the *α*2 domain with an *α*1 domain absent; one in gene cluster 3.2 contained only an *α*3 domain; and one in gene cluster 4.1 contained an additional *α*3 domain with the absence of an *α*1 domain. All the nine loci had high GC contents ranging from 58.1% to 64.3%.

#### Phylogenetic inference

**Figure 4:**
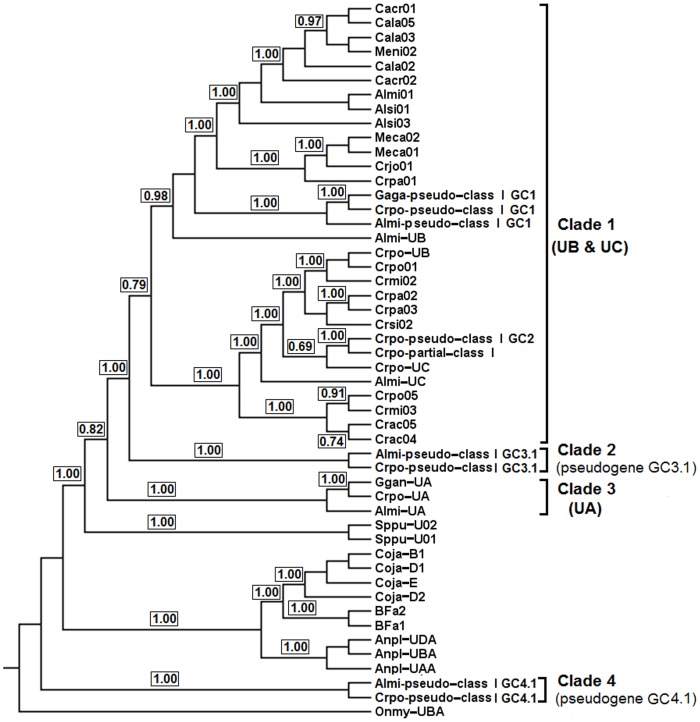
Bayesian phylogenetic tree of MHC class I genes. The fish MHC class I sequence (*Onmy-UBA*; AF287487) is used as an outgroup. Brackets on the right show Clades 1 to 4 of the MHC genes/pseudogenes from Crocodylia identified in the current study and previous publications as described in Materials and Methods. For Clades 1 and 3, gene lineages are named with ‘*U*’ for unknown families of MHC class I and then the locus name, following Klein et al. [86]. Support on branches is indicated by posterior probabilities (PP = 0–1).

Bayesian inference of MHC class I genes from the current study and those from the previous study described in S3 Table showed that they clustered into four clades (Clades 1–4; PP = 0.79–1.00) when a fish sequence (*Oncorhynchus mykiss*) was used as an outgroup (Fig. 4). All these clades contained MHC genes/pseudogenes from the saltwater crocodile, American alligator and/or Indian gharial identified herein. Clades 2–4 each clustered MHC genes/pseudogenes (PP = 1.00) that were assigned to the same MHC locus in the three crocodilian taxa studied. Therefore, orthologous relationships could be suggested for these three clades corresponding to the cluster 3.2 pseudogene, *UA* gene and cluster 4.1 pseudogene respectively, with between-clade pairwise distance ranging from 0.466 to 0.703 showing great divergence between them. Cluster 4.1 pseudogenes from the saltwater crocodile and American alligator within Clade 4 showed dramatic divergence from the rest of MHC genes in Crocodylia analysed here with pairwise genetic distance of exons ranging from 0.564 to 0.744. These MHC class I pseudogenes formed a paraphyletic clade with currently analysed MHC genes from crocodilians, fowl, and the tuatara (*Sphenodon punctatus*), indicating that they are expected to predate the divergence of these vertebrate groups. However, Clade 1 clustered five loci of saltwater crocodile MHC class I identified here (the *UB* gene, *UC* gene, cluster 1 pseudogene, cluster 2 pseudogene and partial MHC class I gene) and all previously sequenced variants (PP = 0.79). This clustering indicated that the Clade 1 variants corresponded to *UB* and *UC* gene lineages, where their loci were detected at different sites on the saltwater crocodile genome. Since the topology in Clade 1 did not allow a clear subdivision of these two genes, we proposed this clade as a representation of *UB* and *UC* gene lineages. The saltwater crocodile *UB* gene clustered well (PP = 1.00) with other five MHC class I sequences from four species of crocodiles (the saltwater crocodile, mugger crocodile, Philippine crocodile and Siamese crocodile), suggesting that they may represent orthologs to the *UB* gene in the saltwater crocodile.

### MHC class II genes

#### Gene structure and content

Six MHC class II loci were identified within gene clusters 5 and 6 (S5 Figure). Three of these loci contained complete coding sequences, including two encoding for *β* chains (II B assigned as ‘*DAB1 and DAB2*’) and another encoding for an *α* chain (II A assigned as ‘*DAA*’). These MHC class II A and B genes encoded for proteins that were 268 and 257 aa and consisted of four to six exons,: exon 1 encoding the leader peptide, exons 2 and 3 encoding the two extracellular domains (*α*1/*α*2 or *β*1/*β*2 domains) and exons 4–6 encoding the transmembrane domain and cytoplasmic tail. They contained 100% conserved aa positions for disulfide bridge-forming (C–C), peptide-binding of antigen N and C termini and/or CD4^+^ binding, suggesting that they were functional. Homology searches with American alligator cDNA sequences appeared to support immunological function of these genes with significant matches between alligator class II A transcript and *DAA* (E-value  = 0.0, aa identity  = 98%, query coverage  = 100%), as well as between alligator class II B transcript and *DAB* (*DAB1* = 2e–175, 85%, 100%; and *DAB2* = 4e–150, 77%, 100%). *DAA* gene showed high similarity to the class II A transcript (aa identity  = 98%) that was found to express in various organs, such as testes, thalamus, spleen, ovary, liver and kidney. The class II B transcript found in tooth, spleen, and stomach, was relatively similar to *DAB1* and *DAB2* genes with aa identity of 85% and 77%, respectively. The remaining three loci, which corresponded to MHC class II B, consisted of *i*) a locus containing a partial coding sequence of leader peptide and *β*1 domain; *ii*) a locus in gene cluster 5 containing a large deletion at the *β*2 domain; and *iii*) a locus in gene cluster 6 that was found to have stop codons at the leader peptide. The first locus was considered to be putatively functional with aa identity of 75% and 52% to *DAB1* and *DAB2*, respectively; the last two loci were pseudogenes.

#### Phylogenetic inference

**Figure 5:**
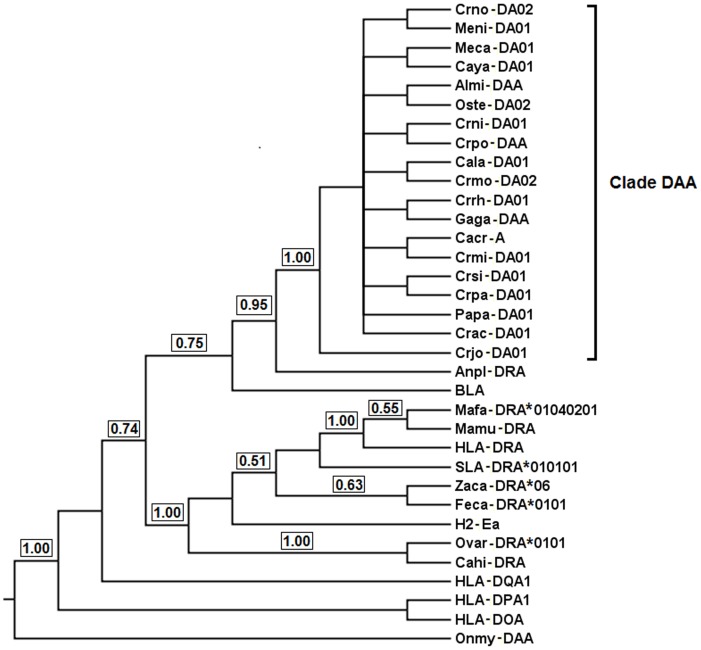
Bayesian phylogenetic tree of MHC class II A genes. The fish MHC class II A sequence (*Onmy-DAA*; AFP94173) is used as an outgroup. A bracket on the right shows the *DAA* gene lineage of the MHC genes from Crocodylia identified in the current study and previous publications as described in Materials and Methods. This gene lineage is named with ‘*DAA*’ (an abbreviation for MHC class II A), following Klein et al. [86]. Support on branches is indicated by posterior probabilities (PP = 0–1).

Bayesian inference of MHC class II A genes identified among the saltwater crocodile, American alligator and Indian gharial studied here and those from other 16 species of Crocodylia in the previous study (S3 Table) showed that they formed a monophyletic clade (PP = 1.0) without clear subdivision in the clade when the fish sequence was used as an outgroup (Fig. 5). All the genes, including *Crpo-DAA*, *Almi-DAA* and *Gaga-DAA* showed high aa identity with an overall genetic distance of 0.004, indicating that they are orthologous to each other. The analysis of introns 1 to 3 among the full-length *DAA* genes identified in the current study was consistent showing little pairwise genetic distance ranging from 0.034–0.057. Using this phylogeny, the *DAA* locus identified on the saltwater crocodile genome enabled us to assign the previously sequenced variants from the other 16 species of Crocodylia to this locus. In addition, all the genes from Crocodylia clustered as a sister clade to mallard and chicken MHC class II A, *Anpl-DRA* and *BLA*, respectively, suggesting that these avian genes are orthologs to *DAA* genes among crocodilians.

**Figure 6:**
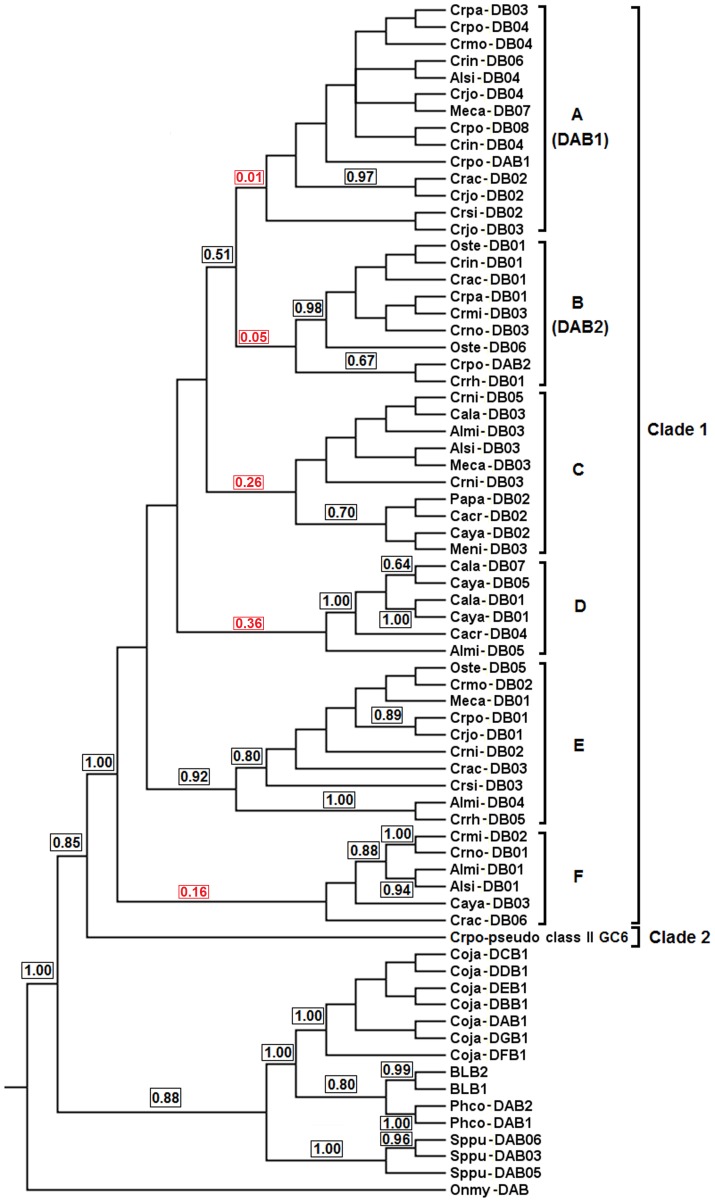
Bayesian phylogenetic analysis of MHC class II B sequences. The fish MHC class II B sequence (*Onmy-DAB*; FR688148) is used as an outgroup. Brackets on the right show Clades 1 and 2 of the MHC variants from Crocodylia, and six subclades (A–F) for Clade 1. For Subclades A and B, gene lineages are named with ‘*DAB*’ (an abbreviation for MHC class II B) and then the identification number, following Klein et al. [86]. Support on branches is indicated by posterior probabilities (PP = 0–1).

Bayesian inference of MHC class II B genes identified in this study and those from the previous study described in S3 Table showed two clades (clades 1 and 2) using the fish sequence as an outgroup (PP = 0.85–1.0; Fig. 6). In Clade 1, the tree was found to have six subclades (1A–1F) with low branch support. Subclades 1A and 1B provided two separate clusterings of the saltwater crocodile *DAB1* and *DAB2* genes, respectively. Subclade 1A clustered the saltwater crocodile *DAB1* gene with MHC variants from six other species of Crocodilidae (crocodiles) and a single species of Alligatoridae (alligators and caimans) (pairwise genetic distance, 0.0–0.074), while Subclade 1B clustered the saltwater crocodile *DAB2* gene with variants from seven other species of Crocodilidae (pairwise genetic distance, 0.0–0.070). These clusterings appear to suggest orthologous relationships of two gene lineages (*DAB1* and *DAB2*) to which the variants correspond. The remaining subclades (1C–1F) contained MHC variants from different species of Crocodylia: three (1C, 1E and 1F) clustered variants from different species of Alligatoridae and Crocodilidae; and one (1D) clustered variants from different species of Alligatoridae. However, collapsing low branch support (PP<0.50) caused Subclades 1A and 1B to cluster together (PP = 0.51) and the others disappeared, except for Subclade 1E (PP = 0.92), suggesting high identity between *DAB1* and *DAB2* genes analysed. In addition, Clade 2 consisted of only a putative pseudogene identified in gene cluster 6, and revealed large divergence to other variants compared, with pairwise genetic distances ranging from 0.425 to 0.507. This could suggest that this pseudogene corresponds to a different locus from the other genes from Crocodylia and may have been selected against in the past resulting in the pseudogenisation of the gene.

### Other genes, retrotransposons and endogenous retrovirus (ERV) sequences

Three novel antigen processing genes (TAP2) were observed near MHC class I genes/pseudogenes within gene cluster 3. A single TAP2 gene (TAP2) provided an intact open reading fragment of 604 aa, and was shown to be expressed due to high sequence identity to unpublished cDNA sequences from the American alligator (TAP2 transcripts 1 and 2; E-value  = 0.0, aa identity  = 87–90%, query coverage  = 90–92%) (S6 Figure). This gene consisted of five exons representing the ABC transporter transmembrane region, and five other exons representing the P-loop containing Nucleoside Triphosphate Hydrolases, and shared significant homology with the reference TAP2 gene (XP_001496065; E-value  = 0.0, aa identity  = 63%, query coverage  = 90%) in the horse (*Equus caballus*). The other two TAP genes were strongly similar to the intact TAP2-like gene in the green anole (XP_003229695; E-value  = 8e–174 for pseudo-TAP2 GC3.1, and 4e–25 for pseudo-TAP2 GC3.2). However, they contained in-frame stop codons and deletions, suggesting that they were pseudogenes. The pseudo-TAP2 in gene cluster 3.2 encompassed 12 exons and had relatively high aa identity (71%) to TAP2 in contrast to the pseudo-TAP2 in gene cluster 3.1, where the ABC transporter transmembrane region were absent and 44% aa identity to the intact TAP2 was observed using seven exon sequences available for the identity analysis. This difference was also true when TAP2 intron sequences were compared with those of pseudo-TAP2 genes in gene clusters 3.1 (identity  = 71.6%) and 3.2 (76.5%), suggesting differences in pseudogenisation between TAP2 genes. In addition, a framework gene TRIM39 was found near the MHC class I pseudogene in gene cluster 4.1. This gene spanned 462 aa and had significant homology to the chicken TRIM39 (NP_001006196; E-value  = 1e–112, aa identity  = 43%, query coverage  = 97%) using BLAST search. The phylogenetic analysis of this homology is shown in S7 Figure. The TRIM gene contained four main domains consistent with the chicken TRIM39 (S8 Figure): RING- zinc finger (aa site 15–61), B-Box-type zinc finger (aa site 91–129), PRY (aa site 288–336), and SPRY (aa site 339–459). This gene was found to be functional due to a match with the cDNA sequence from the American alligator, TRIM transcript (E-value  = 8e–148, aa identity  = 48%, query coverage  = 99%).

A non-MHC gene, actin was also found among the MHC class II genes in gene cluster 6. This gene has functions involved in muscle contraction, cell motility, and cytokinesis [46]. The actin gene identified herein was considered a pseudogene because only a nucleotide binding domain (NBD) was present with a 197-aa region distal to the NBD absent when compared to the unpublished actin cDNA sequence from the American alligator, Actin transcript (S9 Figure). Seventeen following genes corresponding to retrotransposons and ERV were distributed across the MHC gene clusters identified here: 13 retrotransposon reverse transcriptase genes (RT), three Gag-Pol precursor genes, and one non-LTR retrotransposon LINE-1 (Fig. 1). Two retrotransposon RT genes were identified within *UC* and TAP2 genes.

## Discussion

### Size of the saltwater crocodile MHC

The current study shows that MHC in the saltwater crocodile is larger and more complex than other extant archosaurs, (i.e., representative species from the three major groups of birds; S4 and S5 Tables). Firstly in terms of the number of MHC genes, the saltwater crocodile is shown to have multiple copies of MHC class I and II genes/pseudogenes (*n* = 9 and 6, respectively) outnumbering those, especially MHC class I genes, in the minimum essential MHC (*n* = 2 and 5–6, respectively) observed in the Galloansera, such as chickens [9], black grouses [31], and golden pheasants [13]. Similar to the present study, Jaratlerdsiri et al. [40] have demonstrated a large number of MHC class I genes (at least four loci) in the saltwater crocodile. Other galliform species, turkeys and quails, have more elaborate *B* loci than the minimum essential MHC, but still have fewer MHC class I genes than saltwater crocodiles [10], [12], while entire number of MHC class II genes in the crocodile is required for comparisons with that in these two taxa. Considering the number of MHC genes identified in high coverage genome drafts from the remaining two major groups of birds, the Neoaves and Palaeognathae (S5 Table), the complexity of the saltwater crocodile MHC is more evident. The budgerigar (*Melopsittacus undulatus*) [47], peregrine falcon (*Falco peregrinus*) [48], and ostrich (*Struthio camelus*) [49] have fewer MHC class I and II genes (*n* = 1–3 and 1–4, respectively) than the saltwater crocodile. However, there are some passerine birds that also have complex MHC structure with a number of potentially duplicated genes in their genomes [50]–[55]. Supporting this, a genome draft in the zebra finch (*Taeniopygia guttata*; accession number GCA_000151805.2) [11] has 22 and 16 genes in a single chromosome corresponding to MHC class I and II, respectively (S5 Table).

Secondly, the saltwater crocodile MHC genes are longer. It is estimated that the mean length of *UA*, *UB* and *UC* genes identified is about five to six times greater in saltwater crocodiles than that of MHC class I genes in the fowl species described above (∼2 kb on average). Additionally, saltwater crocodile *DAA* and *DAB* genes are approximately two to three times larger than the fowl MHC class II B gene (∼1.8 kb on average) [9], [10], [12], [13], [31]. The differences in gene length seem to be the result of greater intron size observed among the saltwater crocodile MHC genes, as these MHC genes have similar length of coding sequences to published fowl sequences [10], [12], [13], [31]. These findings provide the first evidence of MHC organisation in the saltwater crocodile, contrasting with the compact MHC structure known in the Galloanseres. The Chinese alligator has also shown a well-developed immune defense system with 22 MHC class I genes identified [39]. Evolutionary mechanisms of the differences in MHC size between the saltwater crocodile and birds are discussed below.

### Organisation of the saltwater crocodile MHC

**Figure 7:**
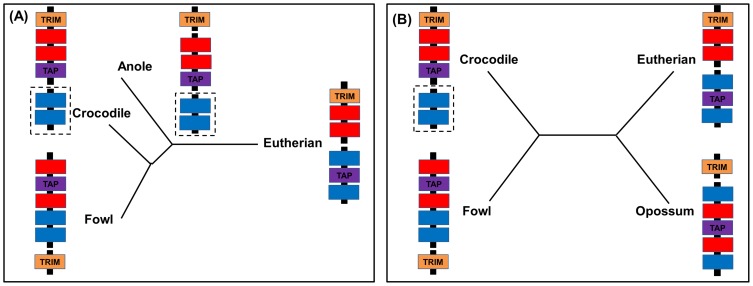
Model of the evolution of the saltwater crocodile MHC. The MHC of crocodiles is compared with that of fowl, eutherians, and anoles (A) or opossums (B). Each coloured box indicates different genes consistent with the legend in Fig. 2. Broken lines indicate absence of linkage between genes; and dashed boxes indicate unknown linkage as a result of unmapped scaffolds in the saltwater crocodile and green anole. MHC gene mapping in fowl (chicken, quail, black grouse, golden pheasant, and turkey), eutherians (human, chimpanzee, gorilla, rat, mouse, dog, cat, cattle, sheep, pig, and horse), green anoles, and opossums is generated using data from Kelley et al. [5], Wang et al. [31], Ye et al. [13], Chaves et al. [12], Wilming et al. [61], Yuhki et al. [92], Gao et al. [14], Gonidez et al. (manuscript in revision), and Belov et al. [15].

The saltwater crocodile MHC (evolution model; Fig. 7A) appears to have a gene organisation intermediate between the fowl *B* locus and the eutherian MHC. The saltwater crocodile shows the same linkage between TAP and MHC class I genes as the fowl (but not eutherians), whilst in contrast TRIM is placed near MHC class I genes in the saltwater crocodile and eutherians (but not fowl). Inclusion of the recently generated green anole MHC in this comparison shows that the gene organisation identified in the saltwater crocodile BACs and the scaffolds from the American alligator and Indian gharial resemble that of the anole with the exception of the close linkage of TRIM and MHC class I among the crocodilians (Godinez et al., manuscript in revision) (Fig. 7A). Given that crocodilians and lizards comprise two major orders of non-avian reptiles (out of 4), it is possible that these immune genes and their organisation, which are conserved between them, also exist across other non-avian reptiles and may suggest putative functional immune/genetic advantages. For instance, the proximity of TAP to MHC class I genes observed in the saltwater crocodile and anole might cause minimal recombination [56], allowing co-evolution between both genes to symbiotically process and present specific peptides consistent with the hypothesis of co-evolving genes observed in the chicken [56]–[58].

Our results with the inclusion of opossum MHC in the model of saltwater crocodile MHC (Fig. 7B) demonstrate that tight linkage of MHC class I and TRIM has preferentially been retained in the saltwater crocodile and eutherians, although the placement of framework genes distant from MHC class I and II regions is found in their sister taxa (fowl and opossums, respectively). The saltwater crocodile MHC class I gene close to TRIM has been maintained after the divergence of crocodilians and birds ∼240 MYA [36], while this structure might have occurred and then maintained in eutherians after the divergence of eutherians and marsupials (∼180 MYA) [15]. Furthermore, this organisation in the saltwater crocodile has also been documented in rodents [25], ungulates [59], [60], and non-human primates [61], [62], as well as other crocodilians studied. Because the main role of the MHC class I and TRIM genes is the same in eliminating infection from viral particles [3], [63] and the proximity of genes within a MHC region has been suggested to allow co-evolution between the genes [56], [57], our finding in organisation between MHC class I and TRIM genes may indicate an advantageous role in their immune function in vertebrates, especially in antiviral immune responses. It could be suggested that TRIM and MHC class I genes in the saltwater crocodile collaborate with each other to eliminate given viral invasion through innate [64] and adaptive immune mechanisms [3], respectively. Based on a study in humans, TRIM proteins can directly bind to certain viral proteins or act as regulators in interferon-induced innate immunity, and thus inhibiting viral maturity and release [65]; some of them are found to significantly interact with DNA-binding proteins to regulate expression of genes within a MHC class I region [66].

### Evolutionary mechanisms of the saltwater crocodile MHC

The additional number of MHC genes and pseudogenes identified in the saltwater crocodile relative to the avian MHC compared above could be a result of the evolutionary mechanisms of gene duplication and loss. Given the multiple copies of MHC genes observed in the saltwater crocodile, and their clustering into various clades and subclades rather than all together as species, it could be suggested that such a mechanism (also known as ‘the birth-and-death process of evolution’) plays a role in complexity of the saltwater crocodile MHC. The effect of gene duplication and loss has previously been documented in mammals and owls to explain orthologous MHC gene relationships [67], [68]. Also, phylogenetic clusterings of these duplicated genes into different clades and subclades suggest that these loci might have evolved independently to each other, allowing them to respond to different environmental pressures or perform novel immune functions. At the same time, the duplicated genes may have been maintained, with similar selective pressures, along with their orthologs from other species of Crocodylia; that is, they all might represent the same biological functions among crocodilians and, therefore, similarity in coding sequences [69]. This is likely to explain why some of the MHC genes from different species are more similar to each other than they are to the duplicated genes within a species. Based on gene loss, some copies of the MHC genes identified in this study have experienced a different fate of evolution, where they were selected against [70] allowing mutations, pseudogenisation, or even deletion from the saltwater crocodile genome. For example, the current clustering of three genomic loci corresponding to MHC class I and II pseudogenes in the saltwater crocodile (ones in gene clusters 3, 4 and 6) into separate clades suggest that they are evolutionarily divergent from their intact gene counterparts.

The identification of retroelements suggests that other mechanisms may have also played a role in creating the additional number of MHC genes in the saltwater crocodile. Kulski et al. [71] suggest that these elements act as insertion and deletion sources on the genome. Long interspersed nuclear element (LINE) sequences identified in the saltwater crocodile, for example, could trigger duplication of genome fragments [72] that contain MHC genes. These duplicated genome fragments then could insert themselves into the saltwater crocodile MHC, leading to expansions of the number of MHC genes and their functions in immunity. This is consistent with that observed in the human MHC, where LINE-mediated duplications have resulted in multiple MHC class I genes, with differing levels of expression, and different expression profiles across tissues [73]. Wan et al. [39] have also shown remarkable abundance of LINE sequences within a genome from Crocodylia (the Chinese alligator genome), and these elements have been suggested to trigger segmental duplications on the genome. In addition, ERV fragments identified might play some role in arrangement of the present-day MHC in the saltwater crocodile consistent with the potential role of Kangaroo endogenous retrovirus (KERV) in the movement of MHC class I genes on the macropod genome [35], [74], [75]. However, the greater gene number observed at the saltwater crocodile MHC relative to the avian MHC may be a result of massive gene loss and subsequent reduction of genome size in birds [30], [76]. Future work investigating complete and contiguous MHC core in the saltwater crocodile is required to refine the size of MHC in this species, as it will provide an opportunity to identify large or small duplicated genome blocks using retroelements as a molecular clock to distinguish those blocks based on differences in their generation times [73].

## Conclusions

Here, we present six MHC gene clusters, representatives of MHC class I and class II regions in the saltwater crocodile genome, providing insights into gene organisation within the two regions. Our comparative analysis shows that the MHC of the saltwater crocodile does not conform to the simple structure found in many birds, and appears to have a distinct organisation to that of avian MHC in relation to the proximity of antigen presentation and framework genes. To extend this knowledge, sequencing of additional positive BACs for MHC markers generated in the current study would be a starting point for full characterisation of the MHC core in the saltwater crocodile genome. Construction and sequencing of cDNA libraries is also needed in this species for accurate gene annotation of the MHC core sequence, as it will be useful for further characterisation of MHC expression and comparative genetics across all vertebrate groups enabling inference of their adaptive immune functions.

## Materials and Methods

### Overgo design

In order to screen the saltwater crocodile genomic BAC library described below, four overgo pairs (forward and reverse) were designed (Table 2) using saltwater crocodile sequences of MHC class I and II from previous studies [40], [42]. The overgos were designed using OligoSpawn software, with a GC content of 50–60% and 36 bp in length (8-bp overlapping) [77]. The specificity of the overgos was checked against vertebrate sequences using the basic local alignment search tool (BLAST; http://www.ncbi.nlm.nih.gov/).

**Table 2:** Four pairs of forward and reverse overgos used for BAC library screening of MHC-associated BACs.

Gene region	Overgo (5′→3′)
MHC class I exon 3	F - GAGAAGACTACATCAGCTATGA
	R – TGTGCGTGCTCGGGTCATAGCT
MHC class II A exon 2	F - TGACTTCGGCAAGTTCACCAGC
	R – CCCTGTGCCTCAAAGCTGGTGA
MHC class II A exon 3	F - GAGGACAACGCTTTCCGCAAGT
	R – GGGCAGGTAGGTGAACTTGCGG
MHC class II B exon 3	F - CGGGATCGAGGTGAAATGGTTG
	R – TCCTGCCCGTTCCTCAACCATT

### BAC library screening

The saltwater crocodile BAC library, Mississippi State University CP_Eba, contained 3.7× genome coverage with 101760 clones [37] that was prepared from a single individual named ‘Errol’ (the University of Sydney Animal Ethics permit number N00/8-2005/3/4177). Errol was a wild-caught adult male housed at Darwin Crocodile Farm near Darwin, Australia. He was housed individually in a purpose-built facility in the tourism section of the farm where trained and experienced staff monitored the animal daily and was fed the equivalent of two whole chickens weekly. During relocation by specialised contractors, blood samples for genome sequencing [38] and BAC library preparation were collected from the cervical sinus as described by Lloyd and Morris [78]. To identify BAC clones with positive MHC inserts, high-density filters containing all BACs in the library were screened with a pool of radiolabeled MHC overgos at the Australian National University (ANU). Each pair of overgos were radioactively labeled with ^32^P-dATP and ^32^P-dCTP (GE Healthcare Life Sciences, Rydalmere NSW) following the BACPAC hybridisation protocol (http://bacpac.chori.org/overgohyb.htm). Unincorporated nucleotides (^32^P-dNTP) were removed using Illustra ProbeQuant G-50 Micro Columns (GE Healthcare Life Sciences, Rydalmere NSW) with the manufacturer’s protocol. These labeled overgos were hybridised onto BAC filters, and washed using the BACPAC hybridisation protocol (http://bacpac.chori.org/overgohyb.htm). Washed filters were exposed to x-ray films, Hyperfilm (GE Healthcare Life Sciences, Rydalmere NSW) for up to ten days at −80°C. The films were developed and positive BAC clones were isolated.

### Secondary screening of MHC-associated BAC clones

The positive BACs were subject to secondary screening using dot blot and each pair of the overgos described above to identify the class of MHC within the BACs and to eliminate false positives. All the BAC clones were cultured at 37°C overnight with Luria Broth (LB) supplemented with 12 µg/ml chloramphenicol. Cultured clones (2 µl) were then applied to gridded Hybond N+ membranes (GE Healthcare Life Sciences, Rydalmere NSW), which were placed on LB agar plates containing 12 µg/ml chloramphenicol, and incubated overnight at 37°C. The membranes were removed from the agar plates, bacterial colonies were lysed, and the DNA was denatured, neutralised and fixed as described by Deakin et al. [79]. The membranes were washed in 6× SSC to eliminate bacterial debris prior to hybridisation. The membranes were screened at 60°C overnight using individual overgo pairs that were radiolabeled, hybridised and washed as described above, and then exposed to Hyperfilm for up to seven days at −80°C. BAC clones positive from the secondary screening were also verified by PCR and direct sequencing, using the appropriate PCR primers and conditions described in the previous publications [40]–[42].

### Library preparation and next-generation sequencing

A subset of BACs (*n* = 9) that showed *i*) very strong signal during all the steps of the BAC screening, and *ii*) clear PCR amplification products and sequences consistent with MHC were selected for next-generation sequencing. Cell culture of BAC clones was performed twice: one at the University of Sydney, Australia where nine positive and verified BACs were incubated overnight at 37°C using LB supplemented with chloramphenicol, and another at Lucigen Corporation (Wisconsin, USA) to which these BACs were submitted for high yields of purified DNA (10 µg). DNA library preparations and 454 sequencing were performed according to internal protocol at Georgia Genomics Facility in Georgia of the USA (http://dna.uga.edu/) using the Roche FLX Genome Sequencer platform, where the GS FLX Titanium Emulsion PCR protocol for the 454 sequencing system (http://www.454.com/) was conducted.

### Data analyses

#### Data control and cleaning

Quality control of the raw 454 reads from each BAC was performed using the following three steps: *i*) removal of duplicated reads (PyroCleaner), *ii*) trimming of adapter and low quality sequences (sff_extract), and *iii*) filtering by read size. The PyroCleaner was used to remove duplicated reads generated by the Roche 454 platform, and to filter sequence data using different criteria, such as length, complexity, and number of undetermined bases [80]. Sff_extract was used to extract information related to read quality and sequences from the sff file and clip multiplex identifier adaptor (MID) at the 5′ end and low quality sequences at the 3′ end of each read (http://bioinf.comav.upv.es/sff_extract/index.html). The last step of data cleaning selected reads from 100 to 800 bp in length. The number of reads per BAC remaining after the quality control and cleaning is shown in S6 Table.

#### Genome assembly and gene annotation

Sequence assembly of the 454 reads cleaned above within each BAC was performed using the following two methods: *i*) *de novo* genome assembly and *ii*) reference genome mapping. Newbler v2.5.3 program, which is designed specifically for *de novo* genome assembly of 454 sequence datasets [81], was used to produce BAC contigs for use in downstream analyses (S7 Table). The reference genome mapping, where the cleaned reads were mapped with reference MHC sequences, was performed using BWA-SW v0.6.1-r104 [82] and BLAT v34 [83] with default parameters. The reference MHC sequences used for this genome mapping consisted of the turkey *B* locus (DQ993255), quail MHC (AB078884), chicken *B* locus (AL023516), and chicken *Y* locus (NC_006103), as they correspond to completely annotated MHC in birds that are sister taxa to crocodilians. Although a number of BAC contigs were constructed using Newbler, mapping the reads against these reference sequences was unsuccessful owing to the fact that small numbers of alignment matches were shown between them (S8 Table).

Contigs within each BAC clone sequenced were subjected to the NCBI genome assembly and annotation pipeline [84]. Based on the NCBI genome assembly, all the contigs within each BAC clone were checked and then trimmed for pIndigoBAC-5 *Hind* III vector sequences used for construction of saltwater crocodile BAC library, using VecScreen (http://www.ncbi.nlm.nih.gov/VecScreen/VecScreen.html). The BAC contigs that contained contaminant sequences from other species were screened using genomic BLAST against genome sequences available in the BLAST database (http://www.ncbi.nlm.nih.gov/sutils/genom_table.cgi). None of these contigs were found to be contaminated in the current study. The resultant BAC contigs without any vector or contaminated sequences were aligned and then merged for construction of larger BAC sequences/scaffolds using MegaBLAST (http://blast.ncbi.nlm.nih.gov/Blast.cgi), where only sequence pairs with more than 80% identity were detected.

The NCBI annotation pipeline was used to annotate our BAC scaffolds using the following two methods: *i*) *ab initio* gene prediction, and *ii*) transcript-based gene modeling [84]. The gene prediction for each scaffold was run using GENSCAN for prediction of coding sequence positions across the BAC sequence [85]. Transcript-based gene models within a BAC scaffold were generated from the best identity hits of nucleotide and amino acid alignments between scaffold and sequences from the RefSeq RNA, RefSeq proteins and American alligator RNA database (http://crocgenomes.org/), and were conducted using standalone NCBI BLAST 2.2.26+ (http://www.ncbi.nlm.nih.gov/books/NBK1762/). Gene annotation from the two methods was merged with preference given to annotation models based on RefSeq RNA sequences or mRNA sequences. Genes predicted by the annotation process were named after the best match to eukaryotic proteins with the exceptions of MHC class I and II genes, which were assigned species-specific nomenclature proposed by Klein et al. [86]. ERV sequences were annotated in the BAC scaffolds using BLAST search against the conserved domain database (http://www.ncbi.nlm.nih.gov/cdd).

#### Comparative analyses of MHC

To conduct comparative MHC analyses between different vertebrate genomes, BAC scaffolds constructed in the saltwater crocodile were compared with reference genomes using CoGe Accelerating Comparative Genomics (http://genomevolution.org/CoGe/). SynMap is a CoGe tool allowing generation of a syntenic dotplot between two genomes and identification of their syntenic regions, collinear sets of genes or regions of sequence similarity [45]. This analysis can also help to link BAC scaffolds into larger fragments using reference genomes from closely related organisms. Reference genomes used for the MHC comparison included those from the American alligator (ID 19558, v0.2.1, unmasked and coding sequence/CDS), Indian gharial (ID 19547, v0.2, unmasked and CDS), red jungle fowl (ID 2731, v2, unmasked and CDS) and human (ID 12142, NCBI v37.2, unmasked and CDS). The reference genomes from two species of Crocodylia were generated using a whole genome sequencing approach, which is different from the BAC-based sequencing conducted in the current study. In addition, organisation of the present saltwater crocodile MHC was compared with genomic regions corresponding to MHC in previous publications from three vertebrate species, including the fugu [87], chicken (AL023516; Shiina et al. [29]) and human (NT_007592). The MHC regions of these species are well-characterised and show unique differences in organisation.

Another CoGe tool, called ‘CoGeBlast’ was used to compare the current BAC scaffolds with the reference genomes at the small scale or gene level for assessment of sequence similarity and gene annotation. This analysis conducts BLAST search, where a query sequence can compare any number of genomes in the CoGe database, and allows visualisation of individual matches in their genomic context [88]. The latest versions of the American alligator (v0.2.1) and Indian gharial reference genomes (v0.2) retrieved from the CoGe database have been poorly sequenced and annotated at the MHC. The tool was used to find conserved genes within MHC regions among the saltwater crocodile and the two other species of Crocodylia and facilitate connection between our neighbouring BAC scaffolds when compared to scaffolds from the two other crocodilians. Novel MHC genes that showed high sequence similarity among the three species of Crocodylia using the BLAST search were annotated with the same names following MHC nomenclature of Klein et al [86]. CoGeBlast results comparing MHC organisations of these species of Crocodylia were displayed using Genome Evolution Analysis (GEvo). This tool helps to compare multiple genomic regions from any number of organisms using a variety of different sequence comparison algorithms in order to quickly identify patterns of genome evolution [88].

#### Phylogenetic and sequence analyses

In order to identify evolutionary lineages within the saltwater crocodile MHC genes, Bayesian inference was conducted using BEAST version 1.5.4 [89]. BEAST performs Markov chain Monte Carlo (MCMC) sampling to estimate posterior probability distributions of parameters involved in substitution, site and clock models as well as tree topology. The best-fitting model of amino acid substitutions was selected using the Bayesian Information Criterion (BIC) and Akaike Information Criterion (AIC) in ModelGenerator version 0.85 [90]. Samples from the posterior probability distribution were drawn every 10^5^ steps over a total of 10^8^ MCMC steps, with the first 10^3^ samples discarded as burn-in. Pairwise genetic distance (*d*) compared the number of nucleotide substitutions or amino acid substitutions in each pair of MHC genes using p-distance and pairwise deletion options in MEGA 5.0 [91].

Bayesian inference described above were performed separately on the three following amino acid sequence datasets that consisted of MHC variants identified within the current BAC sequences and those from Crocodylia and other vertebrates analysed previously [41], [42]: *i*) the MHC class I exons 3 and 4 dataset consisted of eight sequences from the study BACs, 24 from Crocodylia, two from other Reptilia, and nine from Aves, and a single variant from Osteichthyes (outgroup). This dataset had the JTT model with gamma distribution as a best-fit model; *ii*) the MHC class II A exon 2 dataset consisted of a single sequence from the study BACs, 17 from Crocodylia, two from Aves, 12 from Mammalia and a single variant from Osteichthyes (outgroup). This dataset was run with the WAG model with a gamma distribution based on BIC and AIC criteria; and *iii*) the MHC class II B exon 3 dataset consisted of five sequences from the study BACs, 53 from Crocodylia, three from other Reptilia, 11 from Aves, and a single variant from Osteichthyes (outgroup). This dataset fitted the JTT model with gamma distribution. The list of MHC variants and their GenBank accession numbers and species of origin used for phylogenetic comparisons with the current BAC sequences is displayed in S3 Table.

## Supporting Information

S1 Figure **Dot plot analyses across length of saltwater crocodile MHC gene clusters (X axis) and American alligator scaffolds v0.2.1 (Y axis).** Thick lines in the graphs indicate syntenic regions of the two sequences compared with more than 80% identity.
(DOCX)

S2 Figure **Dot plot analyses across length of saltwater crocodile MHC gene clusters (X axis) and Indian gharial scaffolds v0.2 (Y axis).** Thick lines in the graphs indicate syntenic regions of the two sequences compared with more than 80% identity.
(DOCX)

S3 Figure **Syntenic areas (red) between saltwater crocodile TAP2 (MHC gene cluster 3.2 or scaffold-7467 described in** **Table 1** **) and jungle fowl TAP2 (ID 2731, v2, unmasked and CDS).** Gray arrows indicate gene models; green arrows indicate protein coding sequences (CDS); blue arrows (on top of gray genes) indicate mRNA; and yellow arrows indicate approximately 50% GC content in codon wobble positions. Areas highlighted in orange show ambiguous positions.
(DOCX)

S4 Figure **Amino acid alignment of saltwater crocodile MHC class I genes/pseudogenes identified in the current study and a MHC class I transcript from the American alligator.** Variable positions are relative to sequence variants on the top stretching from leader peptide to cytoplasmic domain. Dots represent identical amino acids to the top variant; asterisks represent stop codons; and numbers above the alignments represent the order of amino acid positions. Sites in boxes indicate amino acid positions that are conserved or have expected functions. These amino acid positions also contain the following additional labels: asterisks, conserved peptide-binding residues of antigen N and C termini, as described in Kaufman et al. [93]; diamonds, salt bridge-forming residues; circles, disulfide bridge-forming cysteines; squares: N-glycosylation site; CD8^+^, the expected CD8^+^ binding site. Background colours in the alignments indicate degrees of amino acid identity: 100% in blue; 65-100% in yellow; and below 65% in white.
(DOCX)

S5 Figure **Amino acid alignments of (A) MHC class II A and (B) MHC class II B identified in the current study.** The saltwater crocodile MHC class II genes and pseudogenes were aligned with transcript sequences of MHC class II A and B from the American alligator. Variable positions are relative to sequence variants on the top stretching from leader peptide to cytoplasmic domain. Dots represent identical amino acids to the top variant; asterisks represent stop codons; and numbers above the alignments represent the order of amino acid positions. Sites in boxes indicate amino acid positions that are conserved or have expected functions. These amino acid positions also contain the following additional labels: asterisks, conserved peptide-binding residues of antigen N and C termini, as described in Kaufman et al. [93]; circles, disulfide bridge-forming cysteines; CD4^+^, the expected CD4^+^ binding site. Background colours in the alignments indicate degrees of amino acid identity: 100% in blue; 65–100% in yellow; and below 65% in white.
(DOCX)

S6 Figure **Amino acid alignment of the saltwater crocodile TAP2 gene/pseudogenes identified in the current study and the TAP2 transcripts (1 and 2) observed in the American alligator.** Variable positions are relative to sequence variants on the top. Dots represent identical amino acids to the top variant; asterisks represent stop codons; and numbers above the alignments represent the order of amino acid positions. Background colours in the alignments indicate degrees of amino acid identity: 100% in blue; 65–100% in yellow; and below 65% in white.
(TIF)

S7 Figure **Bayesian phylogenetic tree of TRIM genes from the saltwater crocodile, American alligator and Indian gharial identified in the current study using the human TRIM31 sequence as an outgroup.** Other TRIM genes used for this analysis are from the chicken (AB268588), turkey (DQ993255) and human (NT_007592) as they have MHC cores comprehensively sequenced and annotated. Akaike and Bayesian Information Criteria are used in the ModelGenerator version 0.85 [90] to determine a best-fit substitution model for this phylogenetic analysis. The JTT model with a gamma distribution parameter (alpha), proportion of invariable sites and number of rate categories equivalent to five is selected as the best-fit model. Topological support for the Bayesian Inference is assessed with 1×10^7^ MCMC steps (sampling every 1,000 steps and 1000 burn-in steps) and is indicated by posterior probabilities (PP = 0–1) on branches. Bayesian analysis shows that the TRIM genes from Crocodylia form a monophyletic clade to each other and cluster as sister taxa to TRIM39.1 from the chicken and turkey. It is reasonable to draw a conclusion that these genes identified in the current study are as TRIM39-like genes
(DOCX)

S8 Figure **Amino acid alignment of the saltwater crocodile TRIM39 gene identified in the current study and the TRIM transcript observed in the American alligator.** Variable positions are relative to sequence variants on the top. Dots represent identical amino acids to the top variant; asterisks represent stop codons; and numbers above the alignments represent the order of amino acid positions. Background colours in the alignments indicate degrees of amino acid identity: 100% in blue; 65–100% in yellow; and below 65% in white.
(TIF)

S9 Figure **Amino acid alignment of the saltwater crocodile actin gene identified in the current study and the actin transcript observed in the American alligator.** Variable positions are relative to sequence variants on the top. Dots represent identical amino acids to the top variant; asterisks represent stop codons; and numbers above the alignments represent the order of amino acid positions. Background colours in the alignments indicate degrees of amino acid identity: 100% in blue; 65–100% in yellow; and below 65% in white.
(TIF)

S1 Table **List of 103 BAC clones isolated using dot blot approach for each pair of overgos at the secondary screening of MHC-associated BAC clones and nine BAC clones subjected to BAC-based sequencing.**
(DOCX)

S2 Table **Table. List of novel genes and pseudogenes identified in the saltwater crocodile MHC gene clusters (1–6) and scaffolds from the American alligator and Indian gharial compared in the current study.**
(DOCX)

S3 Table **Table. List of GenBank sequences (MHC class I and II variants) used for the current phylogenetic analyses.**
(DOCX)

S4 Table **Gene copy number of MHC in different bird species (especially Galloanseres/fowl), where their B loci have been fully characterised to date and were used for comparative analyses with the present saltwater crocodile MHC.**
(DOCX)

S5 Table **Predicted MHC class I and II genes within four genome drafts corresponding to two major bird lineages, Neoaves and Palaeognathae.**
(DOCX)

S6 Table **Number of reads validated after three steps of data cleaning among nine BAC clones.**
(DOCX)

S7 Table **Number of BAC contigs among nine BAC clones constructed using Newbler results.**
(DOCX)

S8 Table **Number of aligned sequences and number of hits between read and reference analysed using BWA-SW and BLAT, respectively.**
(DOCX)
