# Supplementary material for: Comparative Genome Analyses Reveal Distinct Structure in the Saltwater Crocodile MHC
Source: PLoS One. 2014 Dec 11;9(12):e114631. doi: 10.1371/journal.pone.0114631 (PMC4263668; doi:10.1371/journal.pone.0114631)
Supplement: S1 Figure — Dot plot analyses across length of saltwater crocodile MHC gene clusters (X axis) and American alligator scaffolds v0.2.1 (Y axis). Thick lines in the graphs indicate syntenic regions of the two sequences compared with more than 80% identity. (DOCX) [file pone.0114631.s001.docx]

**Comparative genome analyses reveal distinct structure in the saltwater crocodile MHC**

PLOS ONE

Weerachai Jaratlerdsiri^1^, Janine Deakin^2,3^, Ricardo Godinez M.^4,14^, Xueyan Shan^5^, Daniel G. Peterson^6^, Sylvain Marthey^7^, Eric Lyons^8^, Fiona M. McCarthy^9^, Sally R. Isberg^1,10^, Damien P. Higgins^1^, Amanda Y. Chong^1^, John St John^11^, Travis C. Glenn^12^, David A. Ray^5,6,13^, Jaime Gongora^1,*^

*^1^ Faculty of Veterinary Science, University of Sydney, Sydney, New South Wales 2006, Australia*

*^2^ Evolution Ecology and Genetics, Research School of Biology, Australian National University, Canberra, Australian Capital Territory 2601, Australia*

*^3^ Institute for Applied Ecology, University of Canberra, Canberra, Australian Capital Territory 2601, Australia*

*^4^ Department of Organismic and Evolutionary Biology, Harvard University, Cambridge, Massachusetts 02138, United States of America*

*^5^ Department of Biochemistry, Molecular Biology, Entomology and Plant Pathology, Mississippi State University, Mississippi State, Mississippi 39762, United States of America*

*^6^ Institute for Genomics, Biocomputing and Biotechnology (IGBB), Mississippi State University, Mississippi State, Mississippi 39762, United States of America*

*^7^ Animal Genetics and Integrative Biology, INRA, UMR 1313 Jouy-en-Josas 78352, France*

*^8^ School of Plant Science, University of Arizona, Tucson, Arizona 85721, United States of America*

*^9^ School of Animal and Comparative Biomedical Sciences, University of Arizona, Tucson, Arizona 85721, United States of America*

*^10^ Center for Crocodile Research, P.O. Box 329, Noonamah, Northern Territory 0837, Australia*

*^11^ Department of Biomolecular Engineering, University of California, Santa Cruz, California 95064, United States of America*

*^12^ Department of Environmental Health Science, University of Georgia, Athens, Georgia 30602, United States of America*

*^13^ Current Address: Department of Biological Sciences, Texas Tech University, Lubbock, Texas 79409, United States of America*

*^14^ Department of Genetics, Harvard Medical School, 77 Louis Pasteur Ave., Boston, Massachusetts 02115, United States of America*

* Corresponding author: Phone: +61-2 9036 9348. Fax: +61-2 9351 3957. E-mail: [jaime.gongora@sydney.edu.au](mailto:jaime.gongora@sydney.edu.au)


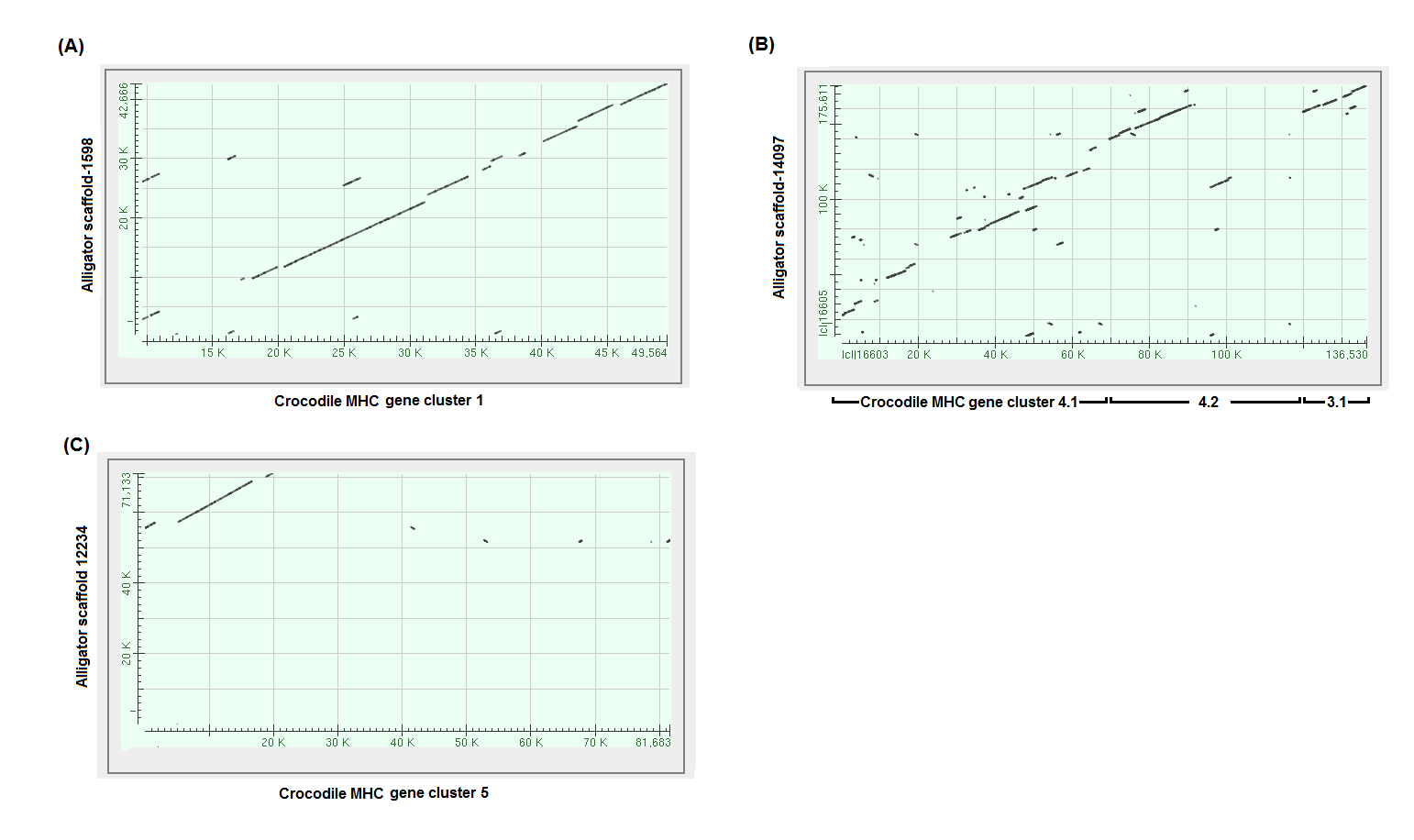


**Figure S1. Dot plot analyses across length of saltwater crocodile MHC gene clusters (X axis) and American alligator scaffolds v0.2.1 (Y axis).** Thick lines in the graphs indicate syntenic regions of the two sequences compared with more than 80% identity
