# Supplementary figures and images for: Comparative Genome Analyses Reveal Distinct Structure in the Saltwater Crocodile MHC
Source: PLoS One. 2014 Dec 11;9(12):e114631. doi: 10.1371/journal.pone.0114631 (PMC4263668; doi:10.1371/journal.pone.0114631)

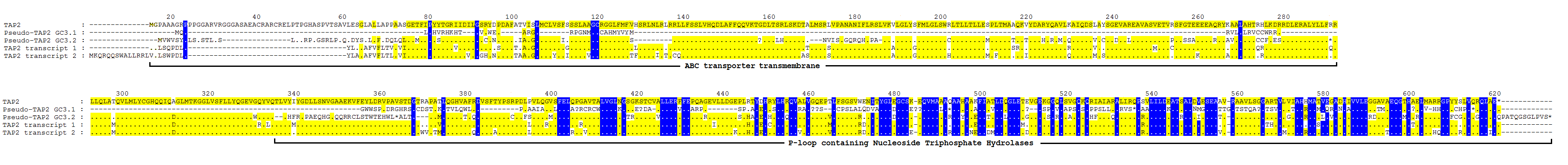

Supplement: S6 Figure — Amino acid alignment of the saltwater crocodile TAP2 gene/pseudogenes identified in the current study and the TAP2 transcripts (1 and 2) observed in the American alligator. Variable positions are relative to sequence variants on the top. Dots represent identical amino acids to the top variant; asterisks represent stop codons; and numbers above the alignments represent the order of amino acid positions. Background colours in the alignments indicate degrees of amino acid identity: 100% in blue; 65–100% in yellow; and below 65% in white. (TIF) [file pone.0114631.s006.tif]

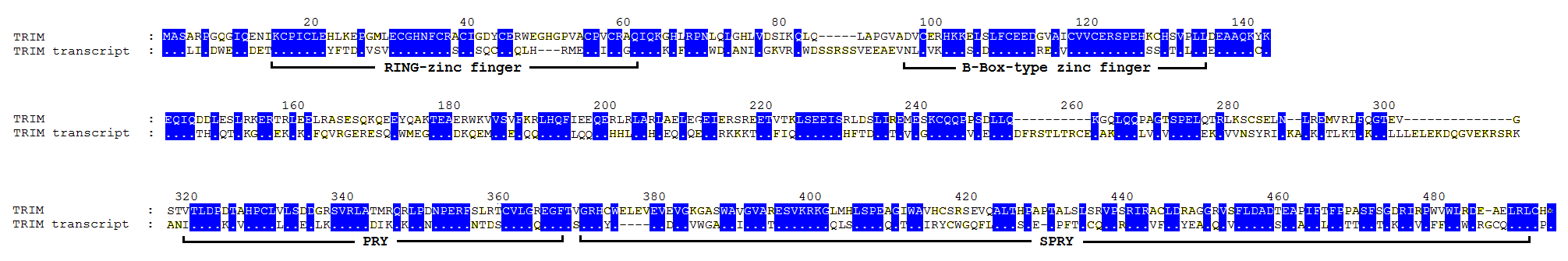

Supplement: S8 Figure — Amino acid alignment of the saltwater crocodile TRIM39 gene identified in the current study and the TRIM transcript observed in the American alligator. Variable positions are relative to sequence variants on the top. Dots represent identical amino acids to the top variant; asterisks represent stop codons; and numbers above the alignments represent the order of amino acid positions. Background colours in the alignments indicate degrees of amino acid identity: 100% in blue; 65–100% in yellow; and below 65% in white. (TIF) [file pone.0114631.s008.tif]

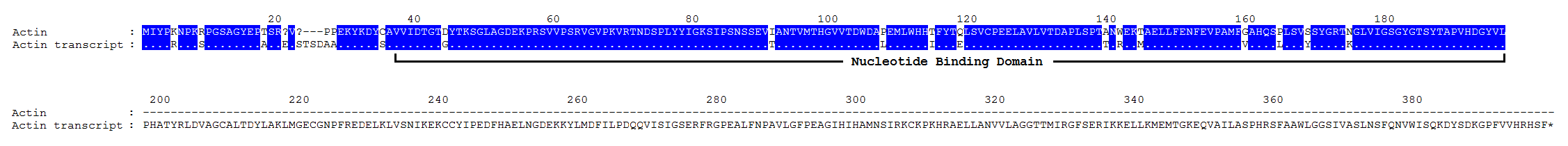

Supplement: S9 Figure — Amino acid alignment of the saltwater crocodile actin gene identified in the current study and the actin transcript observed in the American alligator. Variable positions are relative to sequence variants on the top. Dots represent identical amino acids to the top variant; asterisks represent stop codons; and numbers above the alignments represent the order of amino acid positions. Background colours in the alignments indicate degrees of amino acid identity: 100% in blue; 65–100% in yellow; and below 65% in white. (TIF) [file pone.0114631.s009.tif]
