# Supplementary material for: Comparative Genome Analyses Reveal Distinct Structure in the Saltwater Crocodile MHC
Source: PLoS One. 2014 Dec 11;9(12):e114631. doi: 10.1371/journal.pone.0114631 (PMC4263668; doi:10.1371/journal.pone.0114631)
Supplement: S1 Table — List of 103 BAC clones isolated using dot blot approach for each pair of overgos at the secondary screening of MHC-associated BAC clones and nine BAC clones subjected to BAC-based sequencing. (DOCX) [file pone.0114631.s010.docx]

**Comparative genome analyses reveal distinct structure in the saltwater crocodile MHC**

PLOS ONE

Weerachai Jaratlerdsiri^1^, Janine Deakin^2,3^, Ricardo Godinez M.^4,14^, Xueyan Shan^5^, Daniel G. Peterson^6^, Sylvain Marthey^7^, Eric Lyons^8^, Fiona M. McCarthy^9^, Sally R. Isberg^1,10^, Damien P. Higgins^1^, Amanda Y. Chong^1^, John St John^11^, Travis C. Glenn^12^, David A. Ray^5,6,13^, Jaime Gongora^1,*^

*^1^ Faculty of Veterinary Science, University of Sydney, Sydney, New South Wales 2006, Australia*

*^2^ Evolution Ecology and Genetics, Research School of Biology, Australian National University, Canberra, Australian Capital Territory 2601, Australia*

*^3^ Institute for Applied Ecology, University of Canberra, Canberra, Australian Capital Territory 2601, Australia*

*^4^ Department of Organismic and Evolutionary Biology, Harvard University, Cambridge, Massachusetts 02138, United States of America*

*^5^ Department of Biochemistry, Molecular Biology, Entomology and Plant Pathology, Mississippi State University, Mississippi State, Mississippi 39762, United States of America*

*^6^ Institute for Genomics, Biocomputing and Biotechnology (IGBB), Mississippi State University, Mississippi State, Mississippi 39762, United States of America*

*^7^ Animal Genetics and Integrative Biology, INRA, UMR 1313 Jouy-en-Josas 78352, France*

*^8^ School of Plant Science, University of Arizona, Tucson, Arizona 85721, United States of America*

*^9^ School of Animal and Comparative Biomedical Sciences, University of Arizona, Tucson, Arizona 85721, United States of America*

*^10^ Center for Crocodile Research, P.O. Box 329, Noonamah, Northern Territory 0837, Australia*

*^11^ Department of Biomolecular Engineering, University of California, Santa Cruz, California 95064, United States of America*

*^12^ Department of Environmental Health Science, University of Georgia, Athens, Georgia 30602, United States of America*

*^13^ Current Address: Department of Biological Sciences, Texas Tech University, Lubbock, Texas 79409, United States of America*

*^14^ Department of Genetics, Harvard Medical School, 77 Louis Pasteur Ave., Boston, Massachusetts 02115, United States of America*

* Corresponding author: Phone: +61-2 9036 9348. Fax: +61-2 9351 3957. E-mail: [jaime.gongora@sydney.edu.au](mailto:jaime.gongora@sydney.edu.au)

**Table S1.** List of 103 BAC clones isolated using dot blot approach for each pair of overgos at the secondary screening of MHC-associated BAC clones and nine BAC clones subjected to BAC-based sequencing

| **Gene region** | **Secondary screening** | **BAC-based sequencing^a^** |
| --- | --- | --- |
| MHC class I  exon 3 | P7N12, P9O17, P12F13, P27K15, P32E24, P32H12, P33M9, P40B18, P44D11, P48K24, P51L22, P52G19, P55C24, P60B14, P76L5, P77H5, P92F14, P98K11, P99G8, P101F16, P103B4, P117O5, P158P3, P169J10, P177O24, P186J20, P189G14, P192O18, P193A19, P238I8 | **P9O17**, P12F13, **P77H5,** P92F14, P192O18, P193A19 |
| MHC class II A  exon 2 | P41F15, P67G16, P82I19 | **P67G16, P82I19** |
| MHC class II A  exon 3 | P41F15, P57P5, P67G16, P82I19, P85P21, P181L7 | **P67G16, P82I19** |
| MHC class II B  exon 3 | P2F5, P3G2, P4D5, P7N12, P9O17, P10I15, P11N12, P14E10, P15B10, P18E17, P20A18, P21K12, P24M23, P26D14, P31D5, P32E24, P33M9, P37B16, P41F15, P45N7, P45N8, P57P5, P58B19, P60B14, P67G16, P68E9, P68J15, P69I23, P74P17, P76L5, P77H5, P81N11, P82I19, P82K15, P85M13, P85O2, P85P21, P90J16, P102G10, P104E17, P104E19, P104I21, P105F18, P105I7, P106C15, P109H14, P111L22, P116C23, P116J2, P118D8, P123B24, P123G5, P123N10, P129M21, P134G21, P139E14, P142I10, P145L13, P145O8, P150L17, P150N17, P153I11, P157H15, P159L13, P166C8, P169D5, P169F20, P170P1, P170P2, P172M2, P172M8, P176E14, P183P10, P184L16, P186E12, P186I16, P189G14, P193N8, P196C10, P203L5, P218G2, P238I8 | **P9O17, P67G16**, **P77H5**, **P82I19,** P186I16 |

Note – the BAC library consists of 101760 BAC clones. After primary screening of the BAC library, 169 clones were positive with a pool of overgos generated in the current study (Table 2)

^a^ Identity of BAC clones in bold indicates clones that were positive for more than one pair of overgos (or gene region) from the current study
