# Supplementary material for: Comparative Genome Analyses Reveal Distinct Structure in the Saltwater Crocodile MHC
Source: PLoS One. 2014 Dec 11;9(12):e114631. doi: 10.1371/journal.pone.0114631 (PMC4263668; doi:10.1371/journal.pone.0114631)
Supplement: S2 Table — Table. List of novel genes and pseudogenes identified in the saltwater crocodile MHC gene clusters (1–6) and scaffolds from the American alligator and Indian gharial compared in the current study. (DOCX) [file pone.0114631.s011.docx]

**Comparative genome analyses reveal distinct structure in the saltwater crocodile MHC**

PLOS ONE

Weerachai Jaratlerdsiri^1^, Janine Deakin^2,3^, Ricardo Godinez M.^4,14^, Xueyan Shan^5^, Daniel G. Peterson^6^, Sylvain Marthey^7^, Eric Lyons^8^, Fiona M. McCarthy^9^, Sally R. Isberg^1,10^, Damien P. Higgins^1^, Amanda Y. Chong^1^, John St John^11^, Travis C. Glenn^12^, David A. Ray^5,6,13^, Jaime Gongora^1,*^

*^1^ Faculty of Veterinary Science, University of Sydney, Sydney, New South Wales 2006, Australia*

*^2^ Evolution Ecology and Genetics, Research School of Biology, Australian National University, Canberra, Australian Capital Territory 2601, Australia*

*^3^ Institute for Applied Ecology, University of Canberra, Canberra, Australian Capital Territory 2601, Australia*

*^4^ Department of Organismic and Evolutionary Biology, Harvard University, Cambridge, Massachusetts 02138, United States of America*

*^5^ Department of Biochemistry, Molecular Biology, Entomology and Plant Pathology, Mississippi State University, Mississippi State, Mississippi 39762, United States of America*

*^6^ Institute for Genomics, Biocomputing and Biotechnology (IGBB), Mississippi State University, Mississippi State, Mississippi 39762, United States of America*

*^7^ Animal Genetics and Integrative Biology, INRA, UMR 1313 Jouy-en-Josas 78352, France*

*^8^ School of Plant Science, University of Arizona, Tucson, Arizona 85721, United States of America*

*^9^ School of Animal and Comparative Biomedical Sciences, University of Arizona, Tucson, Arizona 85721, United States of America*

*^10^ Center for Crocodile Research, P.O. Box 329, Noonamah, Northern Territory 0837, Australia*

*^11^ Department of Biomolecular Engineering, University of California, Santa Cruz, California 95064, United States of America*

*^12^ Department of Environmental Health Science, University of Georgia, Athens, Georgia 30602, United States of America*

*^13^ Current Address: Department of Biological Sciences, Texas Tech University, Lubbock, Texas 79409, United States of America*

*^14^ Department of Genetics, Harvard Medical School, 77 Louis Pasteur Ave., Boston, Massachusetts 02115, United States of America*

* Corresponding author: Phone: +61-2 9036 9348. Fax: +61-2 9351 3957. E-mail: [jaime.gongora@sydney.edu.au](mailto:jaime.gongora@sydney.edu.au)

**Table S2.** List of novel genes and pseudogenes identified in the saltwater crocodile MHC gene clusters (1-6) and scaffolds from the American alligator and Indian gharial compared in the current study

| **MHC gene clusters / scaffold ID** | **Gene/ pseudogene** | **Saltwater crocodile** | **American alligator** | **Indian gharial** |
| --- | --- | --- | --- | --- |
| 1 | MHC class I pseudogene | √ | ? | ? |
|  | *UA* | √ | √ | √ |
|  | *UB* | √ | √ | ? |
| 2 | MHC class I pseudogene | √ | × | × |
| 3.1 | MHC class I (partial) | ? | ? | × |
|  | TAP2 pseudogene | √ | √ | × |
|  | MHC class I pseudogene | √ | √ | × |
| 3.2 | TAP2 pseudogene | √ | × | √ |
|  | MHC class I pseudogene | √ | × | √ |
|  | TAP2 | √ | × | × |
| 4.1 | TRIM39 | √ | √ | ? |
|  | MHC class I pseudogene | √ | √ | ? |
| 4.2 | *UC* | √ | ? | × |
| 5 | *DAA* | √ | √ | √ |
|  | *DAB1* | √ | × | × |
|  | MHC class II B pseudogene | √ | × | × |
|  | MHC class II B (partial) | ? | × | × |
| 6 | Actin | √ | × | × |
|  | *DAB2* | √ | × | × |
|  | MHC class II B pseudogene | √ | × | × |
| S12234^a^ | *DMA* | × | √ | × |
|  | BRD2 | × | √ | × |

Note – Ticks indicate presence of genes or pseudogenes in the species studied; crosses indicate absence of genes or pseudogenes in the species studied; and question marks indicate presence of genes or pseudogenes with incomplete sequences in the species studied due to ambiguous nucleotide sites (N) or the end of scaffold sequences

^a^ Scaffold-12234 of the American alligator genome (ID 19558, v0.2.1)
