# Supplementary material for: Comparative Genome Analyses Reveal Distinct Structure in the Saltwater Crocodile MHC
Source: PLoS One. 2014 Dec 11;9(12):e114631. doi: 10.1371/journal.pone.0114631 (PMC4263668; doi:10.1371/journal.pone.0114631)
Supplement: S3 Table — Table. List of GenBank sequences (MHC class I and II variants) used for the current phylogenetic analyses. (DOCX) [file pone.0114631.s012.docx]

**Comparative genome analyses reveal distinct structure in the saltwater crocodile MHC**

PLOS ONE

Weerachai Jaratlerdsiri^1^, Janine Deakin^2,3^, Ricardo Godinez M.^4,14^, Xueyan Shan^5^, Daniel G. Peterson^6^, Sylvain Marthey^7^, Eric Lyons^8^, Fiona M. McCarthy^9^, Sally R. Isberg^1,10^, Damien P. Higgins^1^, Amanda Y. Chong^1^, John St John^11^, Travis C. Glenn^12^, David A. Ray^5,6,13^, Jaime Gongora^1,*^

*^1^ Faculty of Veterinary Science, University of Sydney, Sydney, New South Wales 2006, Australia*

*^2^ Evolution Ecology and Genetics, Research School of Biology, Australian National University, Canberra, Australian Capital Territory 2601, Australia*

*^3^ Institute for Applied Ecology, University of Canberra, Canberra, Australian Capital Territory 2601, Australia*

*^4^ Department of Organismic and Evolutionary Biology, Harvard University, Cambridge, Massachusetts 02138, United States of America*

*^5^ Department of Biochemistry, Molecular Biology, Entomology and Plant Pathology, Mississippi State University, Mississippi State, Mississippi 39762, United States of America*

*^6^ Institute for Genomics, Biocomputing and Biotechnology (IGBB), Mississippi State University, Mississippi State, Mississippi 39762, United States of America*

*^7^ Animal Genetics and Integrative Biology, INRA, UMR 1313 Jouy-en-Josas 78352, France*

*^8^ School of Plant Science, University of Arizona, Tucson, Arizona 85721, United States of America*

*^9^ School of Animal and Comparative Biomedical Sciences, University of Arizona, Tucson, Arizona 85721, United States of America*

*^10^ Center for Crocodile Research, P.O. Box 329, Noonamah, Northern Territory 0837, Australia*

*^11^ Department of Biomolecular Engineering, University of California, Santa Cruz, California 95064, United States of America*

*^12^ Department of Environmental Health Science, University of Georgia, Athens, Georgia 30602, United States of America*

*^13^ Current Address: Department of Biological Sciences, Texas Tech University, Lubbock, Texas 79409, United States of America*

*^14^ Department of Genetics, Harvard Medical School, 77 Louis Pasteur Ave., Boston, Massachusetts 02115, United States of America*

* Corresponding author: Phone: +61-2 9036 9348. Fax: +61-2 9351 3957. E-mail: [jaime.gongora@sydney.edu.au](mailto:jaime.gongora@sydney.edu.au)

**Table S3.** List of GenBank sequences (MHC class I and II variants) used for the current phylogenetic analyses

| **Dataset** | **Class** | **Species** | **Variant name** | **Accession**  **no.** |
| --- | --- | --- | --- | --- |
| MHC class I | Reptilia | *Crocodylus porosus* (saltwater crocodile) | *Crpo01* | HQ158304 |
| exons 3 and 4 |  |  | *Crpo05* | HQ158308 |
| [1] |  | *Crocodylus acutus* (American crocodile) | *Crac04* | HQ158328 |
|  |  |  | *Crac05* | HQ158329 |
|  |  | *Crocodylus palustris* (mugger crocodile) | *Crpa01* | HQ158330 |
|  |  |  | *Crpa02* | HQ158331 |
|  |  |  | *Crpa03* | HQ158332 |
|  |  | *Crocodylus siamensis* (Siamese crocodile) | *Crsi02* | HQ158334 |
|  |  | *Crocodylus jonsoni* (freshwater crocodile) | *Crjo01* | HQ158348 |
|  |  | *Crocodylus mindorensis* (Philippine crocodile) | *Crmi02* | HQ158355 |
|  |  |  | *Crmi03* | HQ158356 |
|  |  | *Mecistops cataphractus* (African slender- snouted | *Meca01* | HQ158366 |
|  |  | crocodile) | *Meca02* | HQ158367 |
|  |  | *Alligator mississippiensis* (American alligator) | *Almi01* | HQ158319 |
|  |  | *Alligator sinensis* (Chinese alligator) | *Alsi01* | HQ158339 |
|  |  |  | *Alsi03* | HQ158341 |
|  |  | *Caiman crocodylus* (spectacled caiman) | *Cacr01* | HQ158335 |
|  |  |  | *Cacr02* | HQ158336 |
|  |  | *Caiman latirostris* (broad-snouted caiman) | *Cala02* | HQ158361 |
|  |  |  | *Cala03* | HQ158362 |
|  |  |  | *Cala05* | HQ158364 |
|  |  | *Melanosuchus niger* (black caiman) | *Meni02* | HQ158358 |
|  |  | *Sphenodon punctatus* (tuatara) | *Sppu-U*01* | DQ145788 |
|  |  |  | *Sppu-U*02* | DQ145789 |
|  | Aves | *Gallus gallus* (chicken) | *BFa1* | AL023516 |
|  |  |  | *BFa2* |  |
|  |  | *Coturnix japonica* (quail) | *Coja-B1* | AB078884 |
|  |  |  | *Coja-D1* |  |
|  |  |  | *Coja-D2* |  |
|  |  |  | *Coja-E* |  |
|  |  | *Anas platyrhynchos* (mallard) | *Anpl-UAA* | AY885227 |
|  |  |  | *Anpl-UBA* |  |
|  |  |  | *Anpl-UDA* |  |
|  | Osteichthyes (outgroup) | *Oncorhynchus mykiss* (rainbow trout) | *Onmy-UBA* | AF287487 |
|  |  |  |  |  |
|  |  |  |  |  |
| (Cont.) |  |  |  |  |
|  |  |  |  |  |
| **Dataset** | **Class** | **Species** | **Variant name** | **Accession**  **no.** |
| MHC class IIA | Reptilia | *Crocodylus niloticus* (Nile crocodile) | *Crni-DA01* | GU126929 |
| exons 2 and 3 |  | *Crocodylus acutus* (American crocodile) | *Crac-DA01* | GU126934 |
| [2] |  | *Crocodylus palustris* (mugger crocodile) | *Crpa-DA01* | GU126942 |
|  |  | *Crocodylus siamensis* (Siamese crocodile) | *Crsi-DA01* | GU126944 |
|  |  | *Crocodylus rhombifer* (Cuban crocodile) | *Crrh-DA01* | GU126951 |
|  |  | *Crocodylus jonsoni* (freshwater crocodile) | *Crjo-DA01* | GU126954 |
|  |  | *Crocodylus novaeguineae* (New Guinea crocodile) | *Crno-DA02* | GU126953 |
|  |  | *Crocodylus moreletii* (Morelet's crocodile) | *Crmo-DA02* | GU126938 |
|  |  | *Crocodylus mindorensis* (Philippine crocodile) | *Crmi-DA01* | GU126950 |
|  |  | *Osteolaemus tetraspis* (Dwarf crocodile) | *Oste-DA02* | GU126936 |
|  |  | *Mecistops cataphractus* (African slender- snouted | *Meca-DA01* | GU126931 |
|  |  | crocodile) |  |  |
|  |  | *Paleosuchus palpebrosus* (Cuvier's dwarf caiman) | *Papa-DA01* | GU126941 |
|  |  | *Caiman yacare* (Yacare caiman) | *Caya-DA01* | GU126955 |
|  |  | *Caiman latirostris* (broad-snouted caiman) | *Cala-DA01* | GU126948 |
|  |  | *Melanosuchus niger* (black caiman) | *Meni-DA01* | GU126939 |
|  |  | *Caiman crocodilus* (caiman) | *Cacr-A* | AF256650 |
|  | Aves | *Gallus gallus* (chicken) | *Gaga-BLA* | AY357253 |
|  |  | *Anas platyrhynchos* (mallard) | *Anpl-DRA* | AY905539 |
|  | Mammalia | *Felis catus* (domestic cat) | *Feca-DRA* | EU915361 |
|  |  | *Zalophus californianus* (sea lion) | *Zaca-DRA* | AY491455 |
|  |  | *Sus scrofa* (pig) | *SLA-DRA*01* | DQ883224 |
|  |  | *Capra hircus* (goat) | *Cahi-DRA* | AB008754 |
|  |  | *Ovis aries* (sheep) | *Ovar-DRA* | FM986335 |
|  |  | *Macaca fascicularis* (macaque) | *Mafa-DRA* | AB306651 |
|  |  | *Macaca mulatta* (rhesus monkey) | *Mamu-DRA* | NM_001134298 |
|  |  | *Mus musculus* (mouse) | *H2-Ea* | BC106107 |
|  |  | *Homo sapiens* (human) | *HLA-DRA* | NM_019111 |
|  |  |  | *HLA-DQA1* | NM_002122 |
|  |  |  | *HLA-DOA* | NM_002119 |
|  |  |  | *HLA-DPA1* | NM_033554 |
|  | Osteichthyes (outgroup) | *Oncorhynchus mykiss* (rainbow trout) | *Onmy-DAA* | FR688130 |
|  |  |  |  |  |
|  |  |  |  |  |
|  |  |  |  |  |
| (Cont.) |  |  |  |  |
|  |  |  |  |  |
| **Dataset** | **Class** | **Species** | **Variant name** | **Accession**  **no.** |
| MHC class IIB | Reptilia | *Crocodylus porosus* (saltwater crocodile) | *Crpo-DB01* | GU126912 |
| exon 3 |  |  | *Crpo-DB04* | GU126915 |
| [2] |  |  | *Crpo-DB08* | GU126919 |
|  |  | *Crocodylus niloticus* (Nile crocodile) | *Crni-DB02* | GU126822 |
|  |  |  | *Crni-DB03* | GU126823 |
|  |  |  | *Crni-DB05* | GU126825 |
|  |  | *Crocodylus intermedius* (Orinoco crocodile) | *Crin-DB01* | GU126890 |
|  |  |  | *Crin-DB04* | GU126893 |
|  |  |  | *Crin-DB06* | GU126895 |
|  |  | *Crocodylus acutus* (American crocodile) | *Crac-DB01* | GU126827 |
|  |  |  | *Crac-DB02* | GU126828 |
|  |  |  | *Crac-DB03* | GU126829 |
|  |  |  | *Crac-DB06* | GU126832 |
|  |  | *Crocodylus palustris* (mugger crocodile) | *Crpa-DB01* | GU126833 |
|  |  |  | *Crpa-DB03* | GU126835 |
|  |  | *Crocodylus siamensis* (Siamese crocodile) | *Crsi-DB02* | GU126846 |
|  |  |  | *Crsi-DB03* | GU126847 |
|  |  | *Crocodylus rhombifer* (Cuban crocodile) | *Crrh-DB01* | GU126896 |
|  |  |  | *Crrh-DB05* | GU126900 |
|  |  | *Crocodylus jonsoni* (freshwater crocodile) | *Crjo-DB01* | GU126804 |
|  |  |  | *Crjo-DB02* | GU126805 |
|  |  |  | *Crjo-DB03* | GU126806 |
|  |  |  | *Crjo-DB04* | GU126807 |
|  |  | *Crocodylus novaeguineae* (New Guinea | *Crno-DB01* | GU126909 |
|  |  | crocodile) | *Crno-DB03* | GU126911 |
|  |  | *Crocodylus moreletii* (Morelet's crocodile) | *Crmo-DB02* | GU126921 |
|  |  |  | *Crmo-DB04* | GU126923 |
|  |  | *Crocodylus mindorensis* (Philippine crocodile) | *Crmi-DB02* | GU126810 |
|  |  |  | *Crmi-DB03* | GU126811 |
|  |  | *Osteolaemus tetraspis* (Dwarf crocodile) | *Oste-DB01* | GU126836 |
|  |  |  | *Oste-DB05* | GU126840 |
|  |  |  | *Oste-DB06* | GU126841 |
|  |  | *Mecistops cataphractus* (African slender- snouted | *Meca-DB01* | GU126849 |
|  |  | crocodile) | *Meca-DB03* | GU126851 |
|  |  |  | *Meca-DB07* | GU126855 |
|  |  | *Paleosuchus palpebrosus* (Cuvier's dwarf caiman) | *Papa-DB02* | GU126858 |
|  |  |  |  |  |
| (Cont.) |  |  |  |  |
|  |  |  |  |  |
| **Dataset** | **Class** | **Species** | **Variant name** | **Accession**  **no.** |
| MHC class IIB |  | *Alligator mississippiensis* (American alligator) | *Almi-DB01* | GU126813 |
| exon 3 |  |  | *Almi-DB03* | GU126815 |
| [2] |  |  | *Almi-DB04* | GU126816 |
|  |  |  | *Almi-DB05* | GU126817 |
|  |  | *Alligator sinensis* (Chinese alligator) | *Alsi-DB01* | GU126880 |
|  |  |  | *Alsi-DB04* | GU126883 |
|  |  | *Caiman yacare* (Yacare caiman) | *Caya-DB01* | GU126903 |
|  |  |  | *Caya-DB02* | GU126904 |
|  |  |  | *Caya-DB03* | GU126905 |
|  |  |  | *Caya-DB05* | GU126907 |
|  |  | *Caiman crocodylus* (spectacled caiman) | *Cacr-DB02* | GU126865 |
|  |  |  | *Cacr-DB04* | GU126867 |
|  |  | *Caiman latirostris* (broad-snouted caiman) | *Cala-DB01* | GU126871 |
|  |  |  | *Cala-DB03* | GU126873 |
|  |  |  | *Cala-DB07* | GU126877 |
|  |  | *Melanosuchus niger* (black caiman) | *Meni-DB03* | GU126888 |
|  |  | *Sphenodon punctatus* (tuatara ) | *Sppu-DAB*03* | DQ124235 |
|  |  |  | *Sppu-DAB*05* | DQ124237 |
|  |  |  | *Sppu-DAB*06* | DQ124238 |
|  | Aves | *Gallus gallus* (chicken) | *BLB1* | AL023516 |
|  |  |  | *BLB2* |  |
|  |  | *Coturnix japonica* (quail) | *Coja-DAB1* | AB078884 |
|  |  |  | *Coja-DBB1* |  |
|  |  |  | *Coja-DCB1* |  |
|  |  |  | *Coja-DDB1* |  |
|  |  |  | *Coja-DEB1* |  |
|  |  |  | *Coja-DFB1* |  |
|  |  |  | *Coja-DGB1* |  |
|  |  | *Phasianus colchicus* (pheasant) | *Phco-DAB1* | AJ224349 |
|  |  |  | *Phco-DAB2* | AJ224348 |
|  | Osteichthyes (outgroup) | *Oncorhynchus mykiss* (rainbow trout) | *Onmy-DAB* | OMU20944 |

**References**

1. Jaratlerdsiri W, Isberg SR, Higgins DP, Ho SY, Salomonsen J, et al. (2014) Evolution of MHC class I in the Order Crocodylia. Immunogenetics 66: 53-65.

2. Jaratlerdsiri W, Isberg SR, Higgins DP, Miles LG, Gongora J (2014) Selection and trans-species polymorphism of Major Histocompatibility Complex class II genes in the Order Crocodylia. PLOS ONE 9: e87534.
