# Supplementary material for: Comparative Genome Analyses Reveal Distinct Structure in the Saltwater Crocodile MHC
Source: PLoS One. 2014 Dec 11;9(12):e114631. doi: 10.1371/journal.pone.0114631 (PMC4263668; doi:10.1371/journal.pone.0114631)
Supplement: S4 Table — Gene copy number of MHC in different bird species (especially Galloanseres/fowl), where their B loci have been fully characterised to date and were used for comparative analyses with the present saltwater crocodile MHC. (DOCX) [file pone.0114631.s013.docx]

**Comparative genome analyses reveal distinct structure in the saltwater crocodile MHC**

PLOS ONE

Weerachai Jaratlerdsiri^1^, Janine Deakin^2,3^, Ricardo Godinez M.^4,14^, Xueyan Shan^5^, Daniel G. Peterson^6^, Sylvain Marthey^7^, Eric Lyons^8^, Fiona M. McCarthy^9^, Sally R. Isberg^1,10^, Damien P. Higgins^1^, Amanda Y. Chong^1^, John St John^11^, Travis C. Glenn^12^, David A. Ray^5,6,13^, Jaime Gongora^1,*^

*^1^ Faculty of Veterinary Science, University of Sydney, Sydney, New South Wales 2006, Australia*

*^2^ Evolution Ecology and Genetics, Research School of Biology, Australian National University, Canberra, Australian Capital Territory 2601, Australia*

*^3^ Institute for Applied Ecology, University of Canberra, Canberra, Australian Capital Territory 2601, Australia*

*^4^ Department of Organismic and Evolutionary Biology, Harvard University, Cambridge, Massachusetts 02138, United States of America*

*^5^ Department of Biochemistry, Molecular Biology, Entomology and Plant Pathology, Mississippi State University, Mississippi State, Mississippi 39762, United States of America*

*^6^ Institute for Genomics, Biocomputing and Biotechnology (IGBB), Mississippi State University, Mississippi State, Mississippi 39762, United States of America*

*^7^ Animal Genetics and Integrative Biology, INRA, UMR 1313 Jouy-en-Josas 78352, France*

*^8^ School of Plant Science, University of Arizona, Tucson, Arizona 85721, United States of America*

*^9^ School of Animal and Comparative Biomedical Sciences, University of Arizona, Tucson, Arizona 85721, United States of America*

*^10^ Center for Crocodile Research, P.O. Box 329, Noonamah, Northern Territory 0837, Australia*

*^11^ Department of Biomolecular Engineering, University of California, Santa Cruz, California 95064, United States of America*

*^12^ Department of Environmental Health Science, University of Georgia, Athens, Georgia 30602, United States of America*

*^13^ Current Address: Department of Biological Sciences, Texas Tech University, Lubbock, Texas 79409, United States of America*

*^14^ Department of Genetics, Harvard Medical School, 77 Louis Pasteur Ave., Boston, Massachusetts 02115, United States of America*

* Corresponding author: Phone: +61-2 9036 9348. Fax: +61-2 9351 3957. E-mail: [jaime.gongora@sydney.edu.au](mailto:jaime.gongora@sydney.edu.au)

**Table S4.** Gene copy number of MHC in different bird species (especially Galloanseres/fowl), where their *B* loci have been fully characterised to date and were used for comparative analyses with the present saltwater crocodile MHC

| **Species** | **Position** | **MHC**  **class** | **No. loci** | **Gene** | **Description** | **length**  **(bp)** | **Reference** |
| --- | --- | --- | --- | --- | --- | --- | --- |
| Quail | *B* locus | I | 7 | D1 | Intact gene | 1998 | Shiina et al. (2004) |
| (*Coturnix japonica*) |  |  |  | F | Pseudogene | 224 |  |
|  |  |  |  | G | Pseudogene | 460 |  |
|  |  |  |  | H | Pseudogene | 133 |  |
|  |  |  |  | D2 | Intact gene | 1982 |  |
|  |  |  |  | B1 | Intact gene | 1956 |  |
|  |  |  |  | E | Intact gene | 1968 |  |
|  |  | II | 10 | DGB1 | Intact gene | 1210 |  |
|  |  |  |  | DFB1 | Intact gene | 1191 |  |
|  |  |  |  | DEB1 | Intact gene | 1209 |  |
|  |  |  |  | DDB1 | Intact gene | 1221 |  |
|  |  |  |  | DCB1 | Intact gene | 1206 |  |
|  |  |  |  | DBB1 | Intact gene | 1223 |  |
|  |  |  |  | DAB1 | Intact gene | 1152 |  |
|  |  |  |  | DMA1 | Intact gene | 3656 |  |
|  |  |  |  | DMB2 | Intact gene | 2020 |  |
|  |  |  |  | DMB1 | Intact gene | 2153 |  |
| Turkey | *B* locus | I | 2 | ClassIA1 | Intact gene | 2018 | Chaves et al. (2009) |
| (*Meleagris gallopavo*) |  |  |  | ClassIA2 | Intact gene | 2018 |  |
|  |  | II | 6 | ClassIIB1 | Intact gene | 1327 |  |
|  |  |  |  | ClassIIB2 | Intact gene | 1323 |  |
|  |  |  |  | ClassIIB3 | Intact gene | 1383 |  |
|  |  |  |  | DMA | Intact gene | 2328 |  |
|  |  |  |  | DMB1 | Intact gene | 2252 |  |
|  |  |  |  | DMB2 | Intact gene | 2812 |  |
| Black grouse | *B* locus | I | 2 | BF1 | Intact gene | 1989 | Wang et al. (2012) |
| (*Tetrao tetrix*) |  |  |  | BF2 | Intact gene | 2009 |  |
|  |  | II | 5 | BLB1 | Intact gene | 1353 |  |
|  |  |  |  | BLB2 | Intact gene | 1383 |  |
|  |  |  |  | DMA | Intact gene | 2145 |  |
|  |  |  |  | DMB1 | Intact gene | 2253 |  |
|  |  |  |  | DMB2 | Intact gene | 2800 |  |
| Golden pheasant | *B* locus | I | 2 | IA1 | Intact gene | 1999 | Ye et al. (2012) |
| (*Chrysolophus pictus*) |  |  |  | IA2 | Intact gene | 2017 |  |
|  |  | II | 6 | IIB1 | Intact gene | 1399 |  |
|  |  |  |  | IIB2 | Intact gene | 1402 |  |
|  |  |  |  | IIB3 | Intact gene | 1412 |  |
|  |  |  |  | DMA | Intact gene | 2295 |  |
|  |  |  |  | DMB1 | Intact gene | 1887 |  |
|  |  |  |  | DMB2 | Intact gene | 2768 |  |
|  |  |  |  |  |  |  |  |
| **(cont.)** |  |  |  |  |  |  |  |
| **Species** | **Position** | **MHC**  **class** | **No. loci** | **Gene** | **Description** | **length**  **(bp)** | **Reference** |
| Chicken | *B* locus | I | 2 | BFa2 | Intact gene | 2510 | Kaufman et al. (1999) |
| (*Gallus gallus*) |  |  |  | BFa1 | Intact gene | 2493 |  |
|  |  | II | 5 | BLB1 | Intact gene | 1661 |  |
|  |  |  |  | BLB2 | Intact gene | 1515 |  |
|  |  |  |  | BMA1 | Intact gene | 2073 |  |
|  |  |  |  | BMB2 | Intact gene | 1821 |  |
|  |  |  |  | BMB1 | Intact gene | 2087 |  |
| Saltwater crocodile | GC1 | I | 9 | Class I | Pseudogene | 8325 | Current study |
| (*Crocodylus porosus*) |  |  |  | UA | Intact gene | 5055 |  |
|  |  |  |  | UB | Intact gene | 9255 |  |
|  | GC2 |  |  | Class I | Pseudogene | 22378 |  |
|  | GC3.1 |  |  | Class I | Pseudogene | 1902 |  |
|  |  |  |  | Class I-partial | Intact gene | 3337 |  |
|  | GC3.2 |  |  | Class I | Pseudogene | 284 |  |
|  | GC4.1 |  |  | Class I | Pseudogene | 16357 |  |
|  | GC4.2 |  |  | UC | Intact gene | 22266 |  |
|  | GC5 | II | 6 | DAA | Intact gene | 2205 |  |
|  |  |  |  | DAB1 | Intact gene | 5945 |  |
|  |  |  |  | Class II | Pseudogene | 6833 |  |
|  |  |  |  | Class II-partial | Intact gene | 731 |  |
|  | GC6 |  |  | DAB2 | Intact gene | 4701 |  |
|  |  |  |  | Class II | Pseudogene | 6804 |  |
|  |  |  |  |  |  |  |  |

**References**

Chaves, L.D., Krueth, S.B. and Reed, K.M. (2009). Defining the turkey MHC: sequence and genes of the B locus. *J Immunol* **183**: 6530-6537.

Kaufman, J., Milne, S., Gobel, T.W., Walker, B.A., Jacob, J.P., Auffray, C., Zoorob, R. and Beck, S. (1999). The chicken B locus is a minimal essential major histocompatibility complex. *Nature* **401**: 923-925.

Shiina, T., Shimizu, S., Hosomichi, K., Kohara, S., Watanabe, S., Hanzawa, K., Beck, S., Kulski, J.K. and Inoko, H. (2004). Comparative genomic analysis of two avian (quail and chicken) MHC regions. *J Immunol* **172**: 6751-6763.

Wang, B., Ekblom, R., Strand, T.M., Portela-Bens, S. and Höglund, J. (2012). Sequencing of the core MHC region of black grouse (*Tetrao tetrix*) and comparative genomics of the galliform MHC. *BMC Genomics* **13**: 553.

Ye, Q., He, K., Wu, S.Y. and Wan, Q.H. (2012). Isolation of a 97-kb minimal essential MHC B locus from a new reverse-4D BAC library of the golden pheasant. *PLOS ONE* **7**: e32154.
