# Supplementary material for: Comparative Genome Analyses Reveal Distinct Structure in the Saltwater Crocodile MHC
Source: PLoS One. 2014 Dec 11;9(12):e114631. doi: 10.1371/journal.pone.0114631 (PMC4263668; doi:10.1371/journal.pone.0114631)
Supplement: S5 Table — Predicted MHC class I and II genes within four genome drafts corresponding to two major bird lineages, Neoaves and Palaeognathae. (DOCX) [file pone.0114631.s014.docx]

**Comparative genome analyses reveal distinct structure in the saltwater crocodile MHC**

PLOS ONE

Weerachai Jaratlerdsiri^1^, Janine Deakin^2,3^, Ricardo Godinez M.^4,14^, Xueyan Shan^5^, Daniel G. Peterson^6^, Sylvain Marthey^7^, Eric Lyons^8^, Fiona M. McCarthy^9^, Sally R. Isberg^1,10^, Damien P. Higgins^1^, Amanda Y. Chong^1^, John St John^11^, Travis C. Glenn^12^, David A. Ray^5,6,13^, Jaime Gongora^1,*^

*^1^ Faculty of Veterinary Science, University of Sydney, Sydney, New South Wales 2006, Australia*

*^2^ Evolution Ecology and Genetics, Research School of Biology, Australian National University, Canberra, Australian Capital Territory 2601, Australia*

*^3^ Institute for Applied Ecology, University of Canberra, Canberra, Australian Capital Territory 2601, Australia*

*^4^ Department of Organismic and Evolutionary Biology, Harvard University, Cambridge, Massachusetts 02138, United States of America*

*^5^ Department of Biochemistry, Molecular Biology, Entomology and Plant Pathology, Mississippi State University, Mississippi State, Mississippi 39762, United States of America*

*^6^ Institute for Genomics, Biocomputing and Biotechnology (IGBB), Mississippi State University, Mississippi State, Mississippi 39762, United States of America*

*^7^ Animal Genetics and Integrative Biology, INRA, UMR 1313 Jouy-en-Josas 78352, France*

*^8^ School of Plant Science, University of Arizona, Tucson, Arizona 85721, United States of America*

*^9^ School of Animal and Comparative Biomedical Sciences, University of Arizona, Tucson, Arizona 85721, United States of America*

*^10^ Center for Crocodile Research, P.O. Box 329, Noonamah, Northern Territory 0837, Australia*

*^11^ Department of Biomolecular Engineering, University of California, Santa Cruz, California 95064, United States of America*

*^12^ Department of Environmental Health Science, University of Georgia, Athens, Georgia 30602, United States of America*

*^13^ Current Address: Department of Biological Sciences, Texas Tech University, Lubbock, Texas 79409, United States of America*

*^14^ Department of Genetics, Harvard Medical School, 77 Louis Pasteur Ave., Boston, Massachusetts 02115, United States of America*

* Corresponding author: Phone: +61-2 9036 9348. Fax: +61-2 9351 3957. E-mail: [jaime.gongora@sydney.edu.au](mailto:jaime.gongora@sydney.edu.au)

**Table S5.** Predicted MHC class I and II genes within four genome drafts corresponding to two major bird lineages, Neoaves and Palaeognathae

| **Lineage** | **Species** | **Chromosome/scaffold** | **Gene ID^a^** | **Gene start (bp)** | **Gene end (bp)** | **Length (bp)** | **Homology search^b^** | **E-value** | **Region** |
| --- | --- | --- | --- | --- | --- | --- | --- | --- | --- |
| Neoaves | Zebra finch^c^ | Chromosome Un (175225315 bp) | ENSTGUT00000015812 | 70765920 | 70834629 | 68710 | BF2 | 1.00E-16 | Class I |
|  | (*Taeniopygia guttata*) |  | ENSTGUT00000017296 | 51086513 | 51086992 | 480 | BF2 | 3.00E-10 | Class I |
|  |  |  | ENSTGUT00000016722 | 108533951 | 108535992 | 2042 | BF2 | 2.00E-07 | Class I |
|  |  |  | ENSTGUT00000014819 | 91106228 | 91109540 | 3313 | BF2 | 3.00E-07 | Class I |
|  |  |  | ENSTGUT00000015114 | 94260150 | 94267651 | 7502 | BF2 | 4.00E-07 | Class I |
|  |  |  | ENSTGUT00000016467 | 106384855 | 106385130 | 276 | BF2 | 4.00E-07 | Class I |
|  |  |  | ENSTGUT00000015208 | 148223478 | 148223753 | 276 | BF2 | 5.00E-07 | Class I |
|  |  |  | ENSTGUT00000015623 | 10963744 | 10964407 | 664 | BF2 | 6.00E-07 | Class I |
|  |  |  | ENSTGUT00000014316 | 28825031 | 28827323 | 2293 | BF2 | 8.00E-07 | Class I |
|  |  |  | ENSTGUT00000015212 | 148224308 | 148226331 | 2024 | BF2 | 8.00E-07 | Class I |
|  |  |  | ENSTGUT00000014752 | 90577703 | 90578180 | 478 | BF2 | 3.00E-06 | Class I |
|  |  |  | ENSTGUT00000016329 | 73815220 | 73844890 | 29671 | BF2 | 2.00E-06 | Class I |
|  |  |  | ENSTGUT00000016892 | 46681968 | 46861248 | 179281 | BF2 | 6.00E-06 | Class I |
|  |  |  | ENSTGUT00000016913 | 46765335 | 46878693 | 113359 | BF2 | 6.00E-06 | Class I |
|  |  |  | ENSTGUT00000015583 | 124954309 | 124954785 | 477 | BF2 | 1.00E-05 | Class I |
|  |  |  | ENSTGUT00000014312 | 116480555 | 116507641 | 27087 | BF2 | 2.00E-05 | Class I |
|  |  |  | ENSTGUT00000015728 | 69760033 | 69846994 | 86962 | BF2 | 3.00E-05 | Class I |
|  |  |  | ENSTGUT00000016181 | 104347401 | 104347872 | 472 | BF2 | 4.00E-05 | Class I |
|  |  |  | ENSTGUT00000016070 | 73084048 | 73084525 | 478 | BF2 | 6.00E-05 | Class I |
|  |  |  | ENSTGUT00000016052 | 42292316 | 42292793 | 478 | BF2 | 4.00E-05 | Class I |
|  |  |  | ENSTGUT00000019019 | 121300964 | 121401420 | 100457 | BF2 | 1.00E-04 | Class I |
|  |  |  | TGUT00000017514 | 115905156 | 115907729 | 2574 | BF2 | 9.00E-04 | Class I |
|  |  |  | ENSTGUT00000015504 | 124358551 | 124359347 | 797 | BLB2 | 3.00E-24 | Class II |
|  |  |  | TGUT00000017514 | 115905156 | 115907729 | 2574 | BLB2 | 8.00E-24 | Class II |
|  |  |  | ENSTGUT00000015212 | 148224308 | 148226331 | 2024 | BLB2 | 4.00E-23 | Class II |
|  |  |  | ENSTGUT00000015208 | 148223478 | 148223753 | 276 | BLB2 | 2.00E-22 | Class II |
|  |  |  | ENSTGUT00000017619 | 139935693 | 139939733 | 4041 | BLB2 | 1.00E-14 | Class II |
|  |  |  | ENSTGUT00000016467 | 106384855 | 106385130 | 276 | BLB2 | 4.00E-23 | Class II |
|  |  |  | ENSTGUT00000015114 | 94260150 | 94267651 | 7502 | BLB2 | 1.00E-23 | Class II |
|  |  |  | ENSTGUT00000014316 | 28825031 | 28827323 | 2293 | BLB2 | 9.00E-23 | Class II |
|  |  |  | ENSTGUT00000015240 | 8280324 | 8281266 | 943 | BLB2 | 4.00E-22 | Class II |
|  |  |  | ENSTGUT00000016722 | 108533951 | 108535992 | 2042 | BLB2 | 3.00E-23 | Class II |
|  |  |  | ENSTGUT00000015623 | 10963744 | 10964407 | 664 | BLB2 | 2.00E-22 | Class II |
|  |  |  | ENSTGUT00000014819 | 91106228 | 91109540 | 3313 | BLB2 | 5.00E-23 | Class II |
|  |  |  | ENSTGUT00000017821 | 170160089 | 170160881 | 793 | BLB2 | 1.00E-18 | Class II |
|  |  |  | ENSTGUT00000016255 | 16091112 | 16093004 | 1893 | BLB2 | 8.00E-19 | Class II |
|  |  |  | ENSTGUT00000017817 | 170150536 | 170151740 | 1205 | BLB2 | 4.00E-13 | Class II |
|  |  |  | ENSTGUT00000017351 | 93395 | 93685 | 291 | BMB2 | 5.00E-10 | Class II |
|  |  |  |  |  |  |  |  |  |  |
|  |  |  |  |  |  |  |  |  |  |
|  |  |  |  |  |  |  |  |  |  |
|  |  |  |  |  |  |  |  |  |  |
| **(cont.)** |  |  |  |  |  |  |  |  |  |
|  |  |  |  |  |  |  |  |  |  |
| **Lineage** | **Species** | **Chromosome/scaffold** | **Gene ID^a^** | **Gene start (bp)** | **Gene end (bp)** | **Length (bp)** | **Homology search^b^** | **E-value** | **Region** |
| Neoaves | Budgerigar^d^ | Adam_Phillippy_v6_sli_scf900160256899 (1143 bp) | Mun_R000137 | 396 | 807 | 412 | BF2 | 1.00E-45 | Class I |
|  | (*Melopsittacus undulatus*) | Adam_Phillippy_v6_sli_scf900160260853 (1118 bp) | Mun_R000529 | 276 | 548 | 273 | BLB2 | 2.00E-24 | Class II |
|  |  | Adam_Phillippy_v6_sli_scf900160276889 (1286810 bp) | Mun_R002956 | 214583 | 215271 | 689 | BLB2 | 3.00E-11 | Class II |
|  |  | Adam_Phillippy_v6_sli_scf900160259385 (1706 bp) | Mun_R000376 | 346 | 1459 | 1114 | BMA1 | 1.00E-09 | Class II |
|  |  | Adam_Phillippy_v6_sli_scf900160256967 (9608 bp) | Mun_R000142 | 107 | 1613 | 1507 | BMA1 | 3.00E-25 | Class II |
|  | Peregrine falcon^e^ | scaffold277_1 (80903 bp) | Fpe_R007131 | 59046 | 59680 | 635 | BF2 | 3.00E-49 | Class I |
|  | (*Falco peregrinus*) | scaffold405_1 (1895364 bp) | Fpe_R010242 | 1615128 | 1616407 | 1280 | BF2 | 2.00E-10 | Class I |
|  |  |  | Fpe_R010243 | 1622138 | 1623627 | 1490 | BF2 | 3.00E-08 | Class I |
|  |  | scaffold424_1 (597923 bp) | Fpe_R010688 | 20041 | 21955 | 1915 | BLB2 | 1.00E-35 | Class II |
|  |  |  | Fpe_R010687 | 16611 | 18547 | 1937 | BLB2 | 8.00E-07 | Class II |
|  |  |  | Fpe_R010690 | 46612 | 48709 | 2098 | BLB2 | 7.00E-11 | Class II |
| Palaeognathae | Common ostrich^f^ | scaffold669 (130484 bp) | Sca_R013598 | 86362 | 87176 | 815 | BF2 | 1.00E-30 | Class I |
|  | (*Struthio camelus*) | C14327306 (292 bp) | Sca_R000006 | 34 | 282 | 249 | BLB2 | 2.00E-42 | Class II |
|  |  |  |  |  |  |  |  |  |  |

^a^ Predicted genes based on the current annotation updates in each bird genome draft (see Detailed annotation methods in the GigaScience database or GenBank accession number provided below)

^b^ Protein sequences encoded in four chicken *B* genes (BLB2, BF2, BMA1, and BMB2) were chosen for TBLASTN sequence similarity searches against zebra finch, budgerigar, falcon and ostrich assemblies described below using CoGeBlast with a cut-off of E-value = 0.001. Other chicken MHC class I and II genes in the *B* locus (i.e. BLB1, BF1, and BMB1) were excluded from the analysis, as they showed high identity to their counterparts among the four genes

^c^ *Taeniopygia guttata* (BGI: CoGe ID19545, v1, unmasked, assembly accession – GCA_000151805.2) – High genome coverage (5.5x Sanger)

^d^ *Melopsittacus undulatus* (BGI: CoGe ID16823, v1, unmasked, <http://dx.doi.org/10.5524/100059>) – High genome coverage (16x 454; 7x Illumina)

^e^ *Falco peregrinus* (BGI: CoGe ID16780, v1, unmasked, <http://dx.doi.org/10.5524/101006>) - High genome coverage (137.6x Illumina)

^f^ *Struthio camelus* (BGI: CoGe ID16809, v1, unmasked, <http://dx.doi.org/10.5524/101013>) – High genome coverage (85x Illumina)
