# Supplementary material for: Comparative Genome Analyses Reveal Distinct Structure in the Saltwater Crocodile MHC
Source: PLoS One. 2014 Dec 11;9(12):e114631. doi: 10.1371/journal.pone.0114631 (PMC4263668; doi:10.1371/journal.pone.0114631)
Supplement: S6 Table — Number of reads validated after three steps of data cleaning among nine BAC clones. (DOCX) [file pone.0114631.s015.docx]

**Comparative genome analyses reveal distinct structure in the saltwater crocodile MHC**

PLOS ONE

Weerachai Jaratlerdsiri^1^, Janine Deakin^2,3^, Ricardo Godinez M.^4,14^, Xueyan Shan^5^, Daniel G. Peterson^6^, Sylvain Marthey^7^, Eric Lyons^8^, Fiona M. McCarthy^9^, Sally R. Isberg^1,10^, Damien P. Higgins^1^, Amanda Y. Chong^1^, John St John^11^, Travis C. Glenn^12^, David A. Ray^5,6,13^, Jaime Gongora^1,*^

*^1^ Faculty of Veterinary Science, University of Sydney, Sydney, New South Wales 2006, Australia*

*^2^ Evolution Ecology and Genetics, Research School of Biology, Australian National University, Canberra, Australian Capital Territory 2601, Australia*

*^3^ Institute for Applied Ecology, University of Canberra, Canberra, Australian Capital Territory 2601, Australia*

*^4^ Department of Organismic and Evolutionary Biology, Harvard University, Cambridge, Massachusetts 02138, United States of America*

*^5^ Department of Biochemistry, Molecular Biology, Entomology and Plant Pathology, Mississippi State University, Mississippi State, Mississippi 39762, United States of America*

*^6^ Institute for Genomics, Biocomputing and Biotechnology (IGBB), Mississippi State University, Mississippi State, Mississippi 39762, United States of America*

*^7^ Animal Genetics and Integrative Biology, INRA, UMR 1313 Jouy-en-Josas 78352, France*

*^8^ School of Plant Science, University of Arizona, Tucson, Arizona 85721, United States of America*

*^9^ School of Animal and Comparative Biomedical Sciences, University of Arizona, Tucson, Arizona 85721, United States of America*

*^10^ Center for Crocodile Research, P.O. Box 329, Noonamah, Northern Territory 0837, Australia*

*^11^ Department of Biomolecular Engineering, University of California, Santa Cruz, California 95064, United States of America*

*^12^ Department of Environmental Health Science, University of Georgia, Athens, Georgia 30602, United States of America*

*^13^ Current Address: Department of Biological Sciences, Texas Tech University, Lubbock, Texas 79409, United States of America*

*^14^ Department of Genetics, Harvard Medical School, 77 Louis Pasteur Ave., Boston, Massachusetts 02115, United States of America*

* Corresponding author: Phone: +61-2 9036 9348. Fax: +61-2 9351 3957. E-mail: [jaime.gongora@sydney.edu.au](mailto:jaime.gongora@sydney.edu.au)

**Table S6.** Number of reads validated after three steps of data cleaning among nine BAC clones

| **BAC clone** | **No. reads^a^** | **% duplicated reads** | **No. reads after data cleaning^b^** | | **% No. initial reads^c^** |
| --- | --- | --- | --- | --- | --- |
|  |  |  | **Pyrocleaner** | **Size filtering** |  |
| P9 O17 | 8806 | 38.14 | 5915 | 5915 | 67.17 |
| P12 F13 | 26623 | 40.60 | 17557 | 17557 | 65.94 |
| P67 G16 | 29360 | 40.78 | 19372 | 19372 | 65.98 |
| P77 H5 | 45524 | 40.15 | 30204 | 30204 | 66.34 |
| P82 I19 | 38970 | 41.55 | 25698 | 25697 | 65.94 |
| P92 F14 | 44652 | 39.10 | 32683 | 32683 | 73.19 |
| P186 I16 | 13778 | 40.27 | 9335 | 9335 | 67.75 |
| P192 O18 | 7353 | 37.71 | 4933 | 4933 | 67.08 |
| P193 A19 | 18693 | 41.52 | 12339 | 12339 | 66.00 |

^a^ Number of raw sequence reads

^b^ Number of reads remaining after the sff_extract is the same as that after pyrocleaner, as this cleaning step trims sequence parts, but does not remove reads

^c^ Percentage of reads remaining after a process of quality control and cleaning
