# Supplementary material for: Comparative Genome Analyses Reveal Distinct Structure in the Saltwater Crocodile MHC
Source: PLoS One. 2014 Dec 11;9(12):e114631. doi: 10.1371/journal.pone.0114631 (PMC4263668; doi:10.1371/journal.pone.0114631)
Supplement: S7 Table — Number of BAC contigs among nine BAC clones constructed using Newbler results. (DOCX) [file pone.0114631.s016.docx]

**Comparative genome analyses reveal distinct structure in the saltwater crocodile MHC**

PLOS ONE

Weerachai Jaratlerdsiri^1^, Janine Deakin^2,3^, Ricardo Godinez M.^4,14^, Xueyan Shan^5^, Daniel G. Peterson^6^, Sylvain Marthey^7^, Eric Lyons^8^, Fiona M. McCarthy^9^, Sally R. Isberg^1,10^, Damien P. Higgins^1^, Amanda Y. Chong^1^, John St John^11^, Travis C. Glenn^12^, David A. Ray^5,6,13^, Jaime Gongora^1,*^

*^1^ Faculty of Veterinary Science, University of Sydney, Sydney, New South Wales 2006, Australia*

*^2^ Evolution Ecology and Genetics, Research School of Biology, Australian National University, Canberra, Australian Capital Territory 2601, Australia*

*^3^ Institute for Applied Ecology, University of Canberra, Canberra, Australian Capital Territory 2601, Australia*

*^4^ Department of Organismic and Evolutionary Biology, Harvard University, Cambridge, Massachusetts 02138, United States of America*

*^5^ Department of Biochemistry, Molecular Biology, Entomology and Plant Pathology, Mississippi State University, Mississippi State, Mississippi 39762, United States of America*

*^6^ Institute for Genomics, Biocomputing and Biotechnology (IGBB), Mississippi State University, Mississippi State, Mississippi 39762, United States of America*

*^7^ Animal Genetics and Integrative Biology, INRA, UMR 1313 Jouy-en-Josas 78352, France*

*^8^ School of Plant Science, University of Arizona, Tucson, Arizona 85721, United States of America*

*^9^ School of Animal and Comparative Biomedical Sciences, University of Arizona, Tucson, Arizona 85721, United States of America*

*^10^ Center for Crocodile Research, P.O. Box 329, Noonamah, Northern Territory 0837, Australia*

*^11^ Department of Biomolecular Engineering, University of California, Santa Cruz, California 95064, United States of America*

*^12^ Department of Environmental Health Science, University of Georgia, Athens, Georgia 30602, United States of America*

*^13^ Current Address: Department of Biological Sciences, Texas Tech University, Lubbock, Texas 79409, United States of America*

*^14^ Department of Genetics, Harvard Medical School, 77 Louis Pasteur Ave., Boston, Massachusetts 02115, United States of America*

* Corresponding author: Phone: +61-2 9036 9348. Fax: +61-2 9351 3957. E-mail: [jaime.gongora@sydney.edu.au](mailto:jaime.gongora@sydney.edu.au)

**Table S7.** Number of BAC contigs among nine BAC clones constructed using Newbler results

| **BAC clone** | **No. reads^a^** | **No. contigs** | **% reads in contigs^b^** |
| --- | --- | --- | --- |
| P9 O17 | 5915 | 16 | 80.24 |
| P12 F13 | 17557 | 19 | 83.07 |
| P67 G16 | 19372 | 26 | 84.23 |
| P77 H5 | 30204 | 55 | 80.96 |
| P82 I19 | 25697 | 5 | 94.36 |
| P92 F14 | 32683 | 41 | 90.69 |
| P186 I16 | 9335 | 8 | 79.82 |
| P192 O18 | 4933 | 21 | 90.92 |
| P193 A19 | 12339 | 1 | 81.00 |

^a^ Number of 454 reads cleaned after quality control processes

^b^ Percentage of clean 454 reads constructed into BAC contigs in Column 3
