# Supplementary material for: Comparative Genome Analyses Reveal Distinct Structure in the Saltwater Crocodile MHC
Source: PLoS One. 2014 Dec 11;9(12):e114631. doi: 10.1371/journal.pone.0114631 (PMC4263668; doi:10.1371/journal.pone.0114631)
Supplement: S8 Table — Number of aligned sequences and number of hits between read and reference analysed using BWA-SW and BLAT, respectively. (DOCX) [file pone.0114631.s017.docx]

**Comparative genome analyses reveal distinct structure in the saltwater crocodile MHC**

PLOS ONE

Weerachai Jaratlerdsiri^1^, Janine Deakin^2,3^, Ricardo Godinez M.^4,14^, Xueyan Shan^5^, Daniel G. Peterson^6^, Sylvain Marthey^7^, Eric Lyons^8^, Fiona M. McCarthy^9^, Sally R. Isberg^1,10^, Damien P. Higgins^1^, Amanda Y. Chong^1^, John St John^11^, Travis C. Glenn^12^, David A. Ray^5,6,13^, Jaime Gongora^1,*^

*^1^ Faculty of Veterinary Science, University of Sydney, Sydney, New South Wales 2006, Australia*

*^2^ Evolution Ecology and Genetics, Research School of Biology, Australian National University, Canberra, Australian Capital Territory 2601, Australia*

*^3^ Institute for Applied Ecology, University of Canberra, Canberra, Australian Capital Territory 2601, Australia*

*^4^ Department of Organismic and Evolutionary Biology, Harvard University, Cambridge, Massachusetts 02138, United States of America*

*^5^ Department of Biochemistry, Molecular Biology, Entomology and Plant Pathology, Mississippi State University, Mississippi State, Mississippi 39762, United States of America*

*^6^ Institute for Genomics, Biocomputing and Biotechnology (IGBB), Mississippi State University, Mississippi State, Mississippi 39762, United States of America*

*^7^ Animal Genetics and Integrative Biology, INRA, UMR 1313 Jouy-en-Josas 78352, France*

*^8^ School of Plant Science, University of Arizona, Tucson, Arizona 85721, United States of America*

*^9^ School of Animal and Comparative Biomedical Sciences, University of Arizona, Tucson, Arizona 85721, United States of America*

*^10^ Center for Crocodile Research, P.O. Box 329, Noonamah, Northern Territory 0837, Australia*

*^11^ Department of Biomolecular Engineering, University of California, Santa Cruz, California 95064, United States of America*

*^12^ Department of Environmental Health Science, University of Georgia, Athens, Georgia 30602, United States of America*

*^13^ Current Address: Department of Biological Sciences, Texas Tech University, Lubbock, Texas 79409, United States of America*

*^14^ Department of Genetics, Harvard Medical School, 77 Louis Pasteur Ave., Boston, Massachusetts 02115, United States of America*

* Corresponding author: Phone: +61-2 9036 9348. Fax: +61-2 9351 3957. E-mail: [jaime.gongora@sydney.edu.au](mailto:jaime.gongora@sydney.edu.au)

**Table S8.** Number of aligned sequences and number of hits between read and reference analysed using BWA-SW and BLAT, respectively

| BAC clone | Turkey MHC-B | | Quail MHC | | Chicken MHC-B | | Chicken MHC-Y | |
| --- | --- | --- | --- | --- | --- | --- | --- | --- |
|  | BWA-SW | BLAT | BWA-SW | BLAT | BWA-SW | BLAT | BWA-SW | BLAT |
| P9 O17 | 2 | 7 | 5 | 4 | 7 | 2 | 9 | 12 |
| P12 F13 | 38 | 19 | 17 | 1 | 30 | 9 | 14 | 10 |
| P67 G16 | 23 | 18 | 32 | 33 | 28 | 26 | 11 | 25 |
| P77 H5 | 6 | 19 | 8 | 19 | 11 | 10 | 14 | 21 |
| P82 I19 | 22 | 23 | 27 | 38 | 38 | 38 | 7 | 26 |
| P92 F14 | 3 | 1 | 0 | 5 | 2 | 9 | 0 | 1 |
| P186 I16 | 0 | 3 | 0 | 0 | 4 | 0 | 5 | 1 |
| P192 O18 | 0 | 0 | 0 | 0 | 0 | 0 | 0 | 0 |
| P193 A19 | 0 | 0 | 0 | 0 | 0 | 3 | 0 | 0 |
